# Automated annotation and visualisation of high-resolution spatial proteomic mass spectrometry imaging data using HIT-MAP

**DOI:** 10.1038/s41467-021-23461-w

**Published:** 2021-05-28

**Authors:** G. Guo, M. Papanicolaou, N. J. Demarais, Z. Wang, K. L. Schey, P. Timpson, T. R. Cox, A. C. Grey

**Affiliations:** 1 Mass Spectrometry Hub, University of Auckland, Auckland, New Zealand; 2 School of Biological Sciences, University of Auckland, Auckland, New Zealand; 3 The Garvan Institute of Medical Research and The Kinghorn Cancer Centre, UNSW Sydney, Sydney, NSW Australia; 4 School of Life Sciences, University of Technology Sydney, Sydney, NSW Australia; 5 University of Auckland, School of Biological Sciences, Auckland, New Zealand; 6 Department of Biochemistry, Vanderbilt University, Nashville, TN USA; 7 St Vincent’s Clinical School, Faculty of Medicine, UNSW Sydney, Sydney, NSW Australia

**Keywords:** Peptides, Imaging, Mass spectrometry, Proteome informatics

## Abstract

Spatial proteomics has the potential to significantly advance our understanding of biology, physiology and medicine. Matrix-assisted laser desorption/ionisation mass spectrometry imaging (MALDI-MSI) is a powerful tool in the spatial proteomics field, enabling direct detection and registration of protein abundance and distribution across tissues. MALDI-MSI preserves spatial distribution and histology allowing unbiased analysis of complex, heterogeneous tissues. However, MALDI-MSI faces the challenge of simultaneous peptide quantification and identification. To overcome this, we develop and validate HIT-MAP (High-resolution Informatics Toolbox in MALDI-MSI Proteomics), an open-source bioinformatics workflow using peptide mass fingerprint analysis and a dual scoring system to computationally assign peptide and protein annotations to high mass resolution MSI datasets and generate customisable spatial distribution maps. HIT-MAP will be a valuable resource for the spatial proteomics community for analysing newly generated and retrospective datasets, enabling robust peptide and protein annotation and visualisation in a wide array of normal and disease contexts.

## Abstract (web summary)

MALDI-mass spectrometry imaging (MSI) can reveal the distribution of proteins in tissues but tools for protein identification and annotation are sparse. Here, the authors develop an open-source bioinformatic workflow for false discovery rate-controlled protein annotation and spatial mapping from MALDI-MSI data.

## Introduction

Biological tissues are highly compartmentalised due to their complex and diverse functions^1^. Organs and tissues are typically partitioned into histologically distinct, yet functionally co-dependent sub-regions that exhibit diverse cellular and molecular compositions. Importantly, the unique set of expressed proteins, specific to a particular cell type, location, or place in time and/or space, critically underpins tissue and organ function. Significant changes in these proteomes are typically observed in almost all disease-states^2,3^. While proteomic methods such as liquid-chromatography mass spectrometry (LC–MS) allow for deep global characterisation of these proteomes, they typically do so at the expense of spatial information. Given the heterogeneous nature of the proteome across healthy and diseased tissues^4^, this loss of spatial information can limit our understanding of disease mechanisms. Matrix-assisted laser desorption/ionisation mass spectrometry imaging (MALDI–MSI) is gaining recognition in the study of health and disease as a powerful tool capable of surveying this spatial proteomic complexity in order to unlock insights into physiological and disease mechanism^5–7^.

The core strength of MALDI–MSI is the ability to directly analyse and map the distribution of numerous label-free analytes (such as peptides) within a specimen down to the single-cell scale^8–10^. MALDI–MSI offers greater exploratory capacity over other common approaches such as multiplexed immunostaining^11,12^, which rely on labelling predetermined protein targets, and the availability of validated antibodies for those proteins. The unbiased nature of MALDI–MSI allows for the interrogation of whole proteomes, within the limits of detection/sensitivity, providing a systems-level insight into tissue and organ expression patterns^13^. Furthermore, the integration and co-registration of MALDI–MSI datasets with other established and/or emerging technology platforms (such as routine histology and spatial transcriptomics respectively) will significantly increase our understanding of health and disease through combining complementary, orthogonal data types^14,15^.

However, an issue faced in retaining this crucial spatial information, is that MALDI–MSI encounters a technical limitation associated with determining peptide identity. At present, identification and quantification of peptides are mutually exclusive, primarily due to the typically low amounts of each peptide within a given imaging coordinate. Unlike tandem LC–MS (LC–MS/MS), which fosters high-throughput simultaneous sequencing and identification of enzymatically cleaved and fragmented peptide solutions, current MALDI hardware does not possess such capabilities. Even with the arrival of laser capture microdissection^16^ and single-cell LC–MS approaches^17^, it is still not possible to map individual peptide and protein profiles back to whole tissues and organs at the single-cell level given the need to dissociate tissues. Furthermore, single-cell LC–MS is not able to map extracellular protein distributions, such as the matrisome^18^, reinforcing the need for unbiased global in situ proteomic platforms such as MALDI–MSI.

Currently MALDI–MSI operators have no high-throughput approach to quantify and identify peptides, and typically must choose between the two. Post-acquisition analysis approaches typically include manually exploring spectra, utilising feature *m*/*z* values alone without identification, or cross-referencing spectral datasets to orthogonal databases generated via LC–MS/MS performed on matched tissues, all of which are time-consuming, low-throughput and offer limited functional information.

The rapid proliferation of bioinformatic pipelines available in the omics fields has greatly enhanced the analysis of big data. Analytical software development in the MALDI–MSI space has produced numerous visualisation and spatial analysis programs such as ImageQuest (ThermoFisher Scientific), High-Definition Imaging (Waters) and SCiLS Lab (Bruker). However, tools offering annotation and identification of analytes remain sparse. One recent contribution to the spatial mass spectrometry field is Metaspace [https://metaspace2020.eu]^19^, which was designed to perform metabolite annotation of high-resolution metabolomic MALDI–MSI datasets by cross-referencing spectra to large curated metabolomics databases such as the Human Metabolomics Database [https://hmdb.ca/], as well as LipostarMSI^20^, a platform for lipid, drug and metabolite annotation. To our knowledge, no such similar tool exists for peptide and protein annotation of MALDI–MSI datasets.

To address this, we developed HIT-MAP (High-resolution Informatics Toolbox in MALDI mass-spectrometry imaging Proteomics). HIT-MAP is a platform-independent, freely available, open-source R based pipeline for the automated annotation and visualisation of high-resolution proteomic MALDI–MSI datasets. HIT-MAP integrates common MALDI–MSI file formats into an executable proteomic workflow for peptide and protein annotation via a false discover rate (FDR)-controlled peptide mass fingerprinting (PMF) and protein coverage analysis pipeline. This paper presents both the development and use of HIT-MAP, and its application to the identification, mapping and validation of peptides and proteins, using a normal bovine lens and murine brain tissue as proof-of-principle examples, that would be applicable to a wide scope of applications studying normal and diseased tissues.

## Results

### HIT-MAP minimum system requirements

HIT-MAP is an R package available for download through [https://github.com/MASHUOA/HiTMaP/].

To run the HIT-MAP workflow, a minimum of a four-core CPU equipped with 16GB memory is recommended. A full installation of R (v3.6.2 or later) is also required. The package is run from the command line and can be executed using macOS, Linux and Microsoft Windows operating systems, or is available as a self-contained Docker file containing the necessary run environment [https://hub.docker.com/r/mashuoa/hitmap]. By using the BiocParallel^21^ package, parallel processing can be also be leveraged in either fork mode or socket mode to significantly enhance processing speed. A detailed tutorial of HIT-MAP can be found on Github [https://github.com/MASHUOA/HiTMaP/blob/master/README.md].

### HIT-MAP workflow

**Fig. 1:**
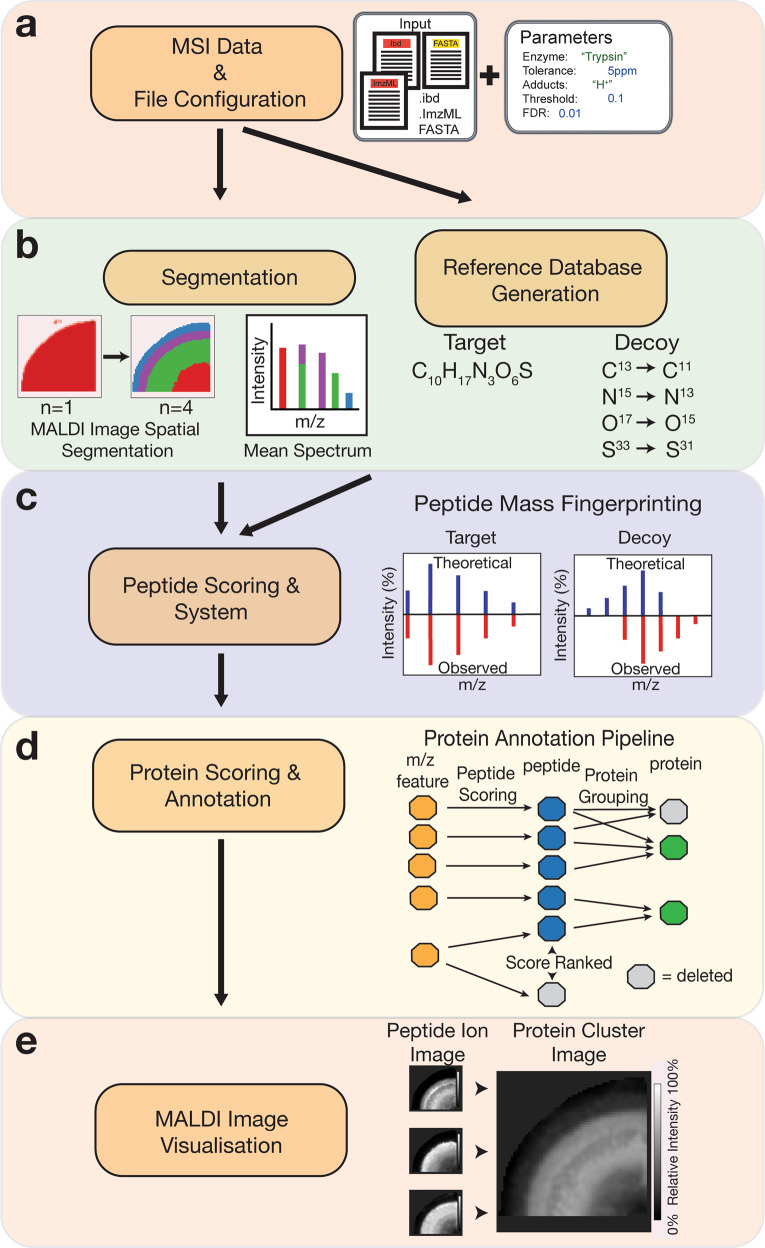
Analysis workflow of HIT-MAP. **a** HIT-MAP utilises as input.imzML and .ibd MALDI imaging datafiles as well as a reference proteome in FASTA format. Parameters including proteolytic enzyme, error tolerance and MALDI image segmentation number are configured in order to represent experimental conditions. **b** [left] Spatial clustering is performed on the MSI dataset producing a mean spectrum to increase the quality of the 2-D mass spectra. [right] The reference proteome is digested in silico to yield a target database to cross-reference to the MSI data, with a corresponding decoy database to statistically control peptide annotation. A series of **c** peptide and **d** protein scoring systems utilising a false-discovery rate target-decoy candidate list statistically controls analyte annotation. **c** Mass features are cross-referenced to the target and decoy databases using a peptide mass fingerprinting approach for peptide annotation, where competing peptides are competitively score-ranked. **d** Protein annotation utilises a peptide grouping strategy that eliminates protein subsets. **e** A customisable visualisation function spatially clusters peptide ion images of a parent protein, representing the summary protein spatial distribution across tissues.

The HIT-MAP workflow is illustrated in (Fig. 1). To begin, HIT-MAP takes as input the commonly utilised MALDI–MSI .imzML file format (Fig. 1a). Both the *.ibd file, which contains the 2-dimensional spectral data, and the *.imzML file, which contains the associated metadata^22^. These files can be exported from both MALDI–MSI data acquisition software (e.g. Bruker’s flexImaging) and analysis software (e.g. Bruker’s SCiLS). It is recommended to export root mean square normalised spectra, in centroid mode, in order to have reliable and robust spatial resolution and signal intensity, although other data normalisation options such as total ion count (TIC) are also compatible. HIT-MAP utilises the Bioconductor package Cardinal^23^ for handling and pre-processing of .imzML data. Importantly, HIT-MAP can be applied to both newly generated, and retrospective complex tissue datasets to facilitate the robust peptide and protein annotation and visualisation in a wide array of normal and disease contexts.

### Signal pre-processing and spatial segmentation

Data pre-processing options include signal smoothing, signal normalisation, baseline reduction, peak picking, and peak alignment. These functions were adopted from the Cardinal package. The user can specify the method to be used in each pre-processing step or skip step(s) by setting the corresponding argument to disable. The user can also set preprocess$force_preprocess to TRUE in order to perform pre-processing regardless of the status of MSI data, and set preprocess$use_preprocessRDS to TRUE to skip the pre-processing and use previously pre-processed data (if available).

To improve *m*/*z* feature detection and subsequent protein annotation, HIT-MAP employs a fully customisable spatial segmentation approach prior to spectrum feature analysis. Given the 2-dimensional nature of MALDI–MSI spectra, clustering segments of MSI imaging data, based on distinct regions of similar spectra, yields averaged spectra for each region that contain a greater number of distinct *m*/*z* features. In addition, *m*/*z* features that may be low in abundance due to their limited spatial distribution will also be enriched with this segmentation approach.

The inherent variability of MALDI ionisation^24^ leads to difficulties in accurately annotating each individual spectrum. Furthermore, while single spectra will generally have higher spectral resolution than mean spectra from clustered regions, single-pixel spectra always contain variability, either induced by ion intensity or natural fluctuation in isotope abundances that will change the profile of an isotopic cluster. Thus, combining the spatially and statistically related pixels into a mean spectrum of these pixels significantly improves *m*/*z* feature detection and subsequent protein annotation. By striking a balance between spectral resolution, signal-to-noise ratio and low abundance feature coverage, HIT-MAP deploys a segmentation approach prior to the spectrum feature analysis to enhance annotation sensitivity and accuracy.

Segmentation in HIT-MAP utilises the Cardinal^23^ package to perform either *K*-means spatial clustering or spatial shrunken centroids clustering in order to segment MALDI–MSI datasets based on region-specific spectral characteristics (Fig. 1b). Cluster number can be manually defined using the spectral_segments_per_file argument in the imaging_identification function using prior knowledge of tissue histology. Of note is that HIT-MAP outputs its spatial segmentation maps that can in themselves be qualitatively cross-referenced with known histology (demonstrated later). Alternatively, this function can be used to reveal the underlying molecular heterogeneity of individual peptide spectra across tissue sections in an exploratory setting and has the ability to identify distinct cellular and molecular compartments based solely on peptide spectra similarity. This segmentation alone is likely to provide a powerful approach for the analysis of diseased tissue states when registered to simple conventional histology images, where there is limited or no prior knowledge of tissue organisation.

HIT-MAP also includes the capability for manual segmentation by the user, through predefining regions of interest (ROI) in target MALDI–MSI datasets and exporting as individual files. The SCiLS software (Bruker) offers the simplest ROI selection and export function whereby *.imzML files for each individual ROI can be exported and analysed separately with data-driven segmentation disabled, by setting the segmentation argument to none. Data converters for various other vendor formats and information on available tools can be found on the MS-Imaging website [https://ms-imaging.org/wp/imzml/software-tools/].

After segmentation of a MALDI–MSI dataset, the HIT-MAP workflow is applied to each segment separately. Mean spectra are obtained from the pixels belonging to each segment. The mass features can be further filtered by using the Threshold argument to ensure their signal-to-noise ratios are eligible for isotopic envelope-based peptide mass fingerprint matching.

### Reference database generation

For each analysis, HIT-MAP generates a customised local database of digested proteolytic peptides in silico (reference database) (Fig. 1b). To generate the reference database for the PMF analysis, HIT-MAP requires a protein sequence file for the complete proteome of the species under investigation in FASTA format. These are readily available from NCBI [https://www.ncbi.nlm.nih.gov/guide/howto/dwn-genome/]. The FASTA file is then handled by the Bioconductor package Biostrings^25^. Alternatively, users may specify a custom-curated library for reference database generation.

HIT-MAP utilises the Bioconductor package Cleaver^26^ to perform an in silico digest of the proteome. Cleaver automatically contains a list of commonly utilised proteolytic enzymes in proteomics applications, and hence common enzyme names can be used as input using the Digestion_site argument. HIT-MAP also offers the option for users to define non-canonical enzymes, provided that both the cleavage site and specificity is known. These may be defined using a regular expression detailing cleavage specificity, along with any exceptions to the cleavage rule. The Bioconductor package protViz^27^ is then used to translate the proteolytic peptide amino acid formulae into precursor masses for cross-referencing to the mass spectra.

HIT-MAP’s customisable digestion framework allows users to tailor reference databases to their need. For example, single or multiple proteases can be specified from a pre-compiled list and in silico digestion performed in parallel or sequential (default) steps. HIT-MAP also offers the option for users to define non-canonical enzymes providing cleavage site and specificity is known. For example, collagenase digestion is a common proteolytic digestion approach used in the analysis of extracellular matrix^28^. The ability to select matrix specific enzyme(s) is designed to complement the emergence of tailored preparation approaches that are customised to specific research needs as well as dual digestion approaches already being used. Finally, HIT-MAP incorporates the ability to specify both fixed and variable modifications (discussed later), allowing for the inclusion of specific post-translational modifications of interest to the researcher.

HIT-MAP also generates a matched decoy database which facilitates FDR-controlled annotation of analytes during scoring. There are three decoy options available to configure using the Decoy_mode argument. Each of these three options was benchmarked and compared, to then define the most suitable approach for peptide assignment as described below. All three are available to the user depending on their specific needs.

The element option will take a given proteolytic peptide from the list of potential targets within the reference database and generate a decoy species via randomly rearranging the elemental composition with equivalent mass. This provides a randomised decoy isotopic pattern for a given target peptide. The disadvantages to this approach are that it can be time-consuming and does not guarantee suitable decoy species in the low mass range.

The adducts option takes a target sequence, where the target candidates contain [H]^+^, [Na]^+^ or [K]^+^ in positive ion mode, and randomly assigns an equal number of highly improbable adducts, such as [He], [Ne], [Ar], [Kr], [Xe] and [Rn]. While this method is effective for annotation of small molecules^19^, a potential issue with this method is the possibility of a candidate peptide with a decoy adduct still matching with high isotopic pattern fidelity to features within the observed spectrum, especially while utilising a global database.

Finally, the isotope option takes the atoms H, O, S, C, N and P creates a reversed isotopic composition for each atom (Fig. 1b, c). Therefore, this method provides an equal number of decoy isotopic patterns to compare to the target isotopic patterns, and also shows robustness against lower-quality spectra. Hence, we recommend using isotope as the default approach.

Once the reference database has been generated, it is saved and can be used as input for additional future analyses by setting the variable use_previous_candidates as TRUE which can reduce processing time substantially.

### Peptide matching and scoring

The analysis pipeline for annotation consists of two sequential peptides (Fig. 1c) and protein-level scoring processes (Fig. 1d). Each process is customisable allowing the user control throughout the analysis pipeline, by using the FDR function, which establishes the statistical threshold for peptide matching and protein annotation.

HIT-MAP utilises the target-decoy database described above for an FDR-controlled strategy in order to statistically control peptide and subsequent protein annotation. In brief, the peptide reference database is generated and serves as the target reference database of all potentially observable peptide species. Within this reference database is a decoy database that establishes a related list of known false positives with a low likelihood of matching to analytes within the observed mass spectra. The combined target-decoy database is cross-referenced to the experimental mass spectra to yield a preliminary list of *m*/*z* features that are then mapped to either target or decoy peptides using an exact mass filtering function. The precision of the exact mass filtering can be configured using the tolerance argument which defines the error in parts per million (ppm) for matching the monoisotopic peak of a species with the observed peak in the experimental spectrum. For high-resolution FT-ICR experiments or similar, it is recommended to input the error determined by the initial instrument calibration of a given experiment if possible.

The preliminary list of matched *m*/*z* features is then scrutinised using the threshold function, which sets a threshold for relative peak intensity in order to exclude noise. Next, a peptide mass fingerprinting analysis in performed, where the rcdk^29^ and rcdklibs^30^ packages simulate a theoretical isotopic pattern for every target and decoy species that matched to an *m*/*z* feature in the spectrum (Fig. 1c).

The SQRTP method contains two terms as indicated in the Intensity_Score formula below. The first term counts the matched peak versus the theoretical peak. Note that peaks below 2.5% of the most intense theoretical isotopic peak are excluded since low abundance theoretical peaks overlap with noise in the observed spectrum and disrupt the scoring. The second term is the square root mean formula characterising how well intensity profiles match between the observed and theoretical isotopic peaks. This results in a scoring algorithm that considers the similarity between the observed and theoretical isotopic patterns factoring in matching peak intensity and mass error, to generate a peptide score using the formula:

1 $$\begin{array}{c}{\mathrm{Intensity}}\_{\mathrm{Score}}=\,\log \left(\frac{{{\mathrm{PeakCount}}}_{{\mathrm{Observed}}}}{{{\mathrm{PeakCount}}}_{{\mathrm{Theoretical}}}}\right) \\ -\,\log \left(\right.\sqrt{\frac{{\sum }_{{{x}}=1}^{{{n}}}({{\mathrm{Theoretical}}}_{{{\mathrm{intensity}}}_{{{x}}}}-\,{{\mathrm{Observed}}}_{{{\mathrm{intensity}}}_{{{x}}}})}{{\sum }_{{{x}}=1}^{{{n}}}{({\mathrm{Theoretical}}\_{{\mathrm{intensity}}}_{{{x}}})}^{2}{({\mathrm{Observed}}\_{{\mathrm{intensity}}}_{{{x}}})}^{2}}}\end{array}$$

2 $${\rm{Mass}}\_{\rm{error}}\_{\rm{Score}}=\left|\left({\rm{p}}\_{\rm{norm}}\_{\rm{dist}}\left(\frac{{\rm{mean}}\_{\rm{ppm}}\_{\rm{error}}}{{\rm{ppm}}\_{\rm{tolerance}}}\right)-0.5\right)\right|$$

3 $$\,{\rm{Pepscore}}={\rm{Intensity}}\_{\rm{Score}}-{\rm{Mass}}\_{\rm{error}}\_{\rm{Score}}$$

It is important to note that it is entirely possible for a *m*/*z* feature to map to more than one peptide in the reference database. Therefore, for each observed *m*/z feature, associated peptides are competitively score-ranked based on their assigned pepscore, allowing for the thresholded elimination of lower-scoring annotations from being included in the subsequent protein annotation steps (Fig. 1d). For users who may wish to evaluate peptide level annotations, this information can be found as an intermediate text file (peptide_SQRTP_ranked.txt saved to each spectrum folder) for a particular input datafile.

### Protein annotation and scoring

Following the annotation of *m*/*z* features with peptide sequences from the reference database and peptide scoring, peptides are then assigned to proteins. It is not possible to conclusively determine peptide sequence based on an MS1 isotopic pattern comparison alone. Instead, HIT-MAP uses protein grouping information to determine the most likely identity of *m*/*z* features, by integrating the commonly utilised strategies for protein grouping^31,32^. Briefly, where a peptide set for Protein A is a subset of the peptide set of Protein B, this will result in Protein B annotation, and Protein A exclusion. However, proteins that share a single peptide and that does not form a subset are removed prior to protein scoring due to the increased statistical power necessary for MALDI–MSI datasets, which may contain a smaller number of peptides and proteins when compared to larger LC–MS/MS datasets (Fig. 1d).

Proteins then enter the protein scoring system which takes a target protein (comprising the set of identified target peptides) and its matched decoy protein (which comprises the set of decoy peptides) and generates a target-decoy system to statistically control protein annotation using the predetermined FDR. The protein score is calculated using the formula:

4 $$\begin{array}{c}{\mathrm{Proscore}}=\frac{{\sum }_{x=1}^{n}({{\mathrm{Pepscore}}}_{x}\ast {\mathrm{log}}({{\mathrm{Intensity}}}_{x}))}{{\mathrm{mean}}({\mathrm{log}}({\mathrm{Intensity}}))}\\ \ast {\mathrm{Protein}}\_{\mathrm{coverage}}\ast {\mathrm{Normalized}}\_{\mathrm{intensity}}\_{\mathrm{factor}}\end{array}$$

Protein_coverage is calculated as the percentage of matched amino acids within a whole protein sequence. Normalised intensity factor is calculated by taking the mean intensity among the peptides of a protein, and normalising by the mean peak intensity value of all peaks in the spectrum. It is used to overcome the low-intensity decoy spectrum matches and enhance the medium to high-intensity peak annotations. The differential scoring of these two sub-databases allows the user to accurately identify annotated peptide sequences and therefore protein identities from their MALDI–MSI datasets.

### Peptide ion image and protein cluster image visualisation

**Fig. 2:**
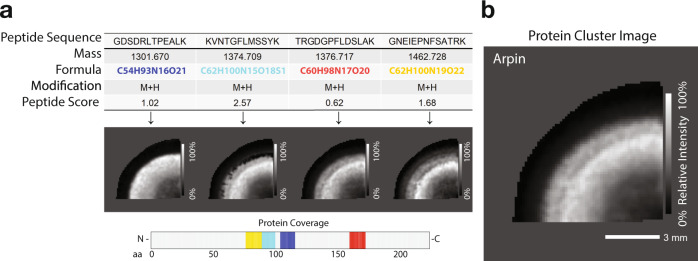
Visualisation of HIT-MAP cluster imaging output in the bovine lens. **a** Example outputs from HIT-MAP visualisation of bovine lens protein arpin, showing the series of peptide data and peptide ion image for a given parent protein, as well as protein coverage (scale bar = 3 mm). **b** Visualisation of the integrated clustering of each individual peptide ion image to demonstrate the overall summary spatial distribution of an annotated protein. Intensity scales represent relative intensity from 0 to 100%. Scale bar = 3 mm.

Following protein annotation, the user has the option to output customisable image maps of both peptide and protein annotation using the plot_cluster_image_grid function (Fig. 1e). The set of peptides for a given parent protein are plotted as well as the *m*/*z* feature from which they originated, with the annotated molecular formula, peptide sequence and peptide score (Fig. 2a). The peptide sequences are then cross-referenced to the parent protein sequence and integrated to visualise total protein coverage on the accompanying plot. HIT-MAP then uses a correlation-based algorithm to plot the summary protein cluster image, which normalises the set of peptide ion images (Fig. 2a) from a given parent protein and integrates them using additive mixing to show a protein spatial distribution revealed by coincident components colours (Fig. 2b). This enhances the intensity of regions with a large spatial overlap of peptide signal. Importantly, HIT-MAP has been designed to plot all matched peptides for a given protein while adding the peptide score in the results table for researchers to assess the relative certainty of each annotated peptide. The application of this function is demonstrated later.

The cluster image plotting has multiple layouts that are appropriate for different situations. For a project with several datafiles, line mode is recommended. In this mode, protein and peptide images of each individual datafile will be rendered in a line and then stacked into a grid to provide a summary view of the project. The grid setting is used to organise images from one datafile into a matrix with user-defined number of columns. The default setting of cluster_color_scale variable is blackwhite to enable the black and white colour scheme in additive mixing mode, although the user is able to choose other colour schemes as detailed in the README.md file. In this mode, components are assigned a grey level only, and the resulting cluster image therefore only represents the sum of all the components as the cluster spatial intensity rather than the spatial coincidence information. The user can also specify the column name that contains the cluster identifier (default setting is Protein) and component identifier (default setting is Peptide). Finally, the output displays the coverage and visualisation of spatial mapping of each peptide within the annotated protein (Fig. 2a), which can then be related to the prior biological knowledge of protein domain structure.

In order to further curate peptide and protein cluster image output, HIT-MAP implements the ability to perform a post-hoc, interquartile range outlier analysis within *m*/*z* bins to filter low scoring peptides, based on their assigned peptide score, from the spatial distribution maps (Supplementary Fig. 1a). This function may be useful if the user wishes to exclude low(er) scoring peptides that could exhibit discordant spatial distributions. By default, HIT-MAP does not implement a stringent spatial concordance filter. This inclusive approach allows the HIT-MAP output to be directly assessed by the user to ultimately interpret the peptide distributions with respect to the tissue-specific biology, whereby apparent spatially discordant peptide distributions may be due to known or relevant biology.

### Output datafiles

For each analysed dataset, two sub-folders are created; one containing all of the identification data for the analysed dataset(s), and the other being a summary folder containing peptide and protein lists as well as the corresponding ion images. Further details on these sub-folders are described in the README.md file. Once an initial analysis is completed, partial re-analysis can be performed by returning to defined points in the HIT-MAP analysis pipeline. Briefly, the user can set the PMF_analysis to FALSE to bypass the peptide mass fingerprint PMF analysis stage. The user can also disable the Protein_feature_summary for the selected datafiles. For example, to render the protein cluster images on already generated datafiles, PMF_analysis is set to FALSE and Protein_feature_summary is set to TRUE. More information on this can also be found in the README.md file.

### Benchmarking of peptide scoring and protein annotation

We initially evaluated HIT-MAP’s peptide identification ability using the commercially available Bruker Peptide Calibration Standard II mixture (Cat #8222570). The peptide calibration standard II mixture contains eight standard peptides ranging from ~700-3500 Da (Table 1). Single spots of 1 µL peptide calibration mix were spotted onto indium tin oxide (ITO) slides and coated with α-Cyano-4-hydroxycinnamic acid (CHCA) in ACN + 0.1% TFA. Slides were then analysed by MALDI–MSI using a Bruker SolariX XR 7T FT-ICR. MALDI–MSI datasets were then exported as *.imzML and *.ibd files from the flexImaging software. The *.imzML and *.ibd files were imported and run through HIT-MAP.

**Table 1:** Characteristics and identification parameters of Bruker’s Peptide Calibrant II.

Name	Sequence	Protein score	Modification	Missed cleavages	[M+H]^+^ monoisotopic
Bradykinin 1–7	RPPGFSP	2.55		1	757.3992
Angiotensin_II	DRVYIHPF	4.51		1	1046.542
Angiotensin_I	DRVYIHPFHL	4.74		1	1296.685
Substance_P	RPKPQQFFGLM	1.28		2	1347.735
Bombesin^a^	XQRLGNQWAVGHLM	1.61	Amide	1	1619.822
ACTH_clip_1–17	SYSMEHFRWGKPVGKKR	2.65		5	2093.086
ACTH_clip_18–39	RPVKVYPNGAEDESAEAFPLEF	2.59		2	2465.198
Somatostatin_28^a^	SANSNPAMAPRERKAGC′KNFFWKTFTSC′	−0.25	Disulfide bond	5	3147.471

^a^X indicates Pyroglutamic acid, C′C′ indicates disulphide bond.

A reference database was generated by tryptic digestion of the *Bos taurus* proteome (Uniprot FASTA format, downloaded 12th August 2019), which was manually curated to contain the additional 8 peptide calibrants as distinct protein entries. A *m*/*z* range of 700 to 4000 was defined, yielding a reference database of 7,787,557 candidates. A *m*/*z* range above 700 ensures that the mapped peptide sequences are generally greater than 7 amino acids in length. An upper threshold of *m*/*z* 4000 was selected since no additional high abundance features are generally found in the range above this value.

Of note, Bombesin contains an X amino acid in the published sequence dataset^33^ for the calibrant denoting a pyroglutamic acid within the sequence. HIT-MAP includes the option for customised amino acid definition using the Substitute_AA argument. This was configured to use the amino acid definition function to set X to the molecular formula of pyroglutamic acid.

**Fig. 3:**
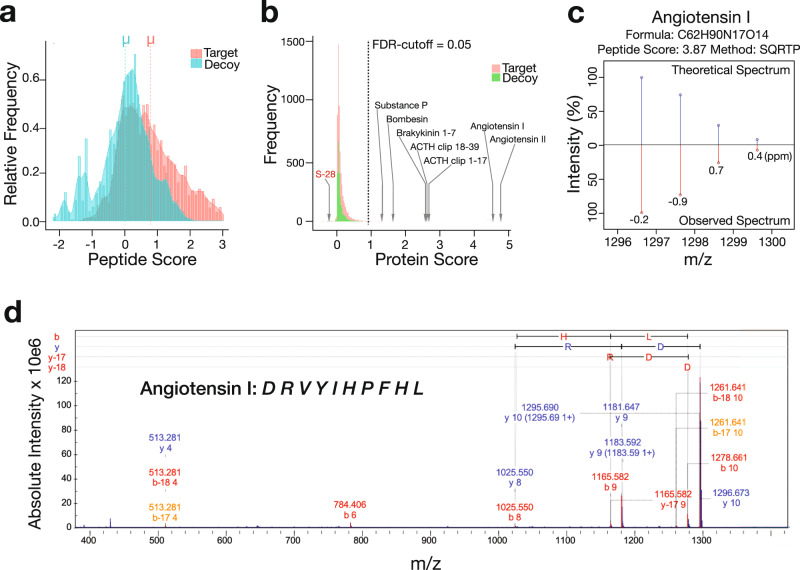
Benchmarking of HIT-MAP against the Bruker Peptide Calibrant II. The frequency distribution of **a** peptide and **b** protein target-decoy candidate list scores demonstrates the robust separation of our false-discovery rate (FDR) system accurately identifying 7/8 peptides. **c** Example of the HIT-MAP predicted and actual peptide mass fingerprint analysis of angiotensin I from the Bruker Peptide Calibrant. **d** Tandem MS/MS validation of angiotensin I identification in Bruker Peptide Calibrant spot. ppm = parts per million.

HIT-MAP analysis of MALDI–MSI datasets obtained from the peptide calibrant spots confirmed statistically significant target-decoy separation (Fig. 3a) confirming our scoring system has the capacity to separate true peptide spectra matched to the in silico generated reference database from the decoy database. Using an error tolerance of 5 ppm and minimum signal intensity of 0.1% for observed spectra, with a standard FDR cut-off of 0.05, 6/8 peptides received a protein score above the FDR cut-off (Protein Score >1) and were accurately annotated (Supplementary Fig. 1b).

Somatostatin-28 appeared to fall outside the FDR cut-off indicating that the theoretical spectra differed significantly from that observed (Supplementary Fig. 1b). Further interrogation revealed the presence of two cysteine amino acids within Somatostatin-28, and we hypothesise that a possible disulphide bond was causing a subtle mass shift leading to it scoring below the FDR cut-off. Initially, bombesin was not annotated (Supplementary Fig. 1b), despite the specific inclusion of pyroglutamic acid as a custom amino acid. However, we hypothesise that this was due to the C-terminal amide modification of bombesin which has been well characterised and leads to a −0.98402 Dalton mass difference^34^.

To deal with modifications such as this, HIT-MAP offers a fixed and variable modification function allowing users to search for protein modifications expected within their experimental or biological set-up. Fixed modifications replace a candidate amino acid in the reference database, while variable modifications create a complementary database. Of note, users should expect longer processing times with an increased number of variable modifications.

To confirm our hypothesis on bombesin, HIT-MAP was re-run using the fixed modifications, disulphide bond and amide, and up to 1 missed cleavage. In this setting, bombesin scored above the protein scoring threshold yielding a total of 7/8 calibration peptides annotated (Fig. 3b). However, Somatostatin-28 still did not meet the FDR cut-off, likely due to the fact that disulphide bond formation only yields a mass shift of 2 Da. In this case, we presume only a proportion of the somatostatin-28 peptides have formed disulphide bonds, leading to an overlap in the isotopic patterns of the two peptide forms and an inability of the mass analyser to resolve the [M+H]^+^ (somatostain-28 with no disulphide bond) and [M+H−2H]^+^ (somatostatin-28 with a disulphide bond) peaks during spectrum acquisition. This small mass shift does not allow sufficient separation of the isotopic peak patterns within the observed mass spectrum during PMF analysis and consequently leads to a low score. Accurately resolving small post-translational mass shifts such as this computationally may not be possible, however biological approaches, such as disulphide bond reduction during experimental preparation that is typically used in LC–MS would overcome this limitation.

In addition to peptide modification, HIT-MAP can account for the presence of different adducts, either naturally occurring, or introduced through preparation workflows. In the peptide calibrant example, only [H]^+^ adducts were considered, since [Na]^+^ adducts were in low abundance. In tissue, the proportion of peptide signal from [Na]^+^ adducts may be higher relative to the [H]^+^ adducts, depending on the tissue preparation procedure and natural salt concentrations in different tissues and tissue regions. However, since [H]^+^ adducts are still likely to be considerably more abundant than [Na]^+^adducts, in this instance, it would be recommended to first run HIT-MAP in [H]^+^ adduct configuration. Based on these results, the user could then run a second HIT-MAP annotation using a reduced database with multiple adducts to increase the coverage of targeted ion species and refine *m*/*z* feature assignments.

Peptide mass fingerprint analysis comparing the theoretical isotopic pattern of target peptides with the isotopic pattern of candidate peptide calibrants demonstrated equivalent peak intensities with minor mass defect (Fig. 3c, Supplementary Fig. 1c), thus facilitating an overall high peptide and subsequent protein score. The presence and identity of peptide calibrants were validated by performing MALDI-FT-ICR-MS/MS on *m*/*z* features following their annotation by HIT-MAP (Fig. 3d and Supplementary Fig. 1c, d), confirming our in silico HIT-MAP annotations.

### HIT-MAP application

To validate HIT-MAP as a robust tool for high-resolution MALDI–MSI imaging peptide annotation, we applied it to a previously published MALDI–MSI fresh frozen, normal bovine lens dataset^35^.

**Fig. 4:**
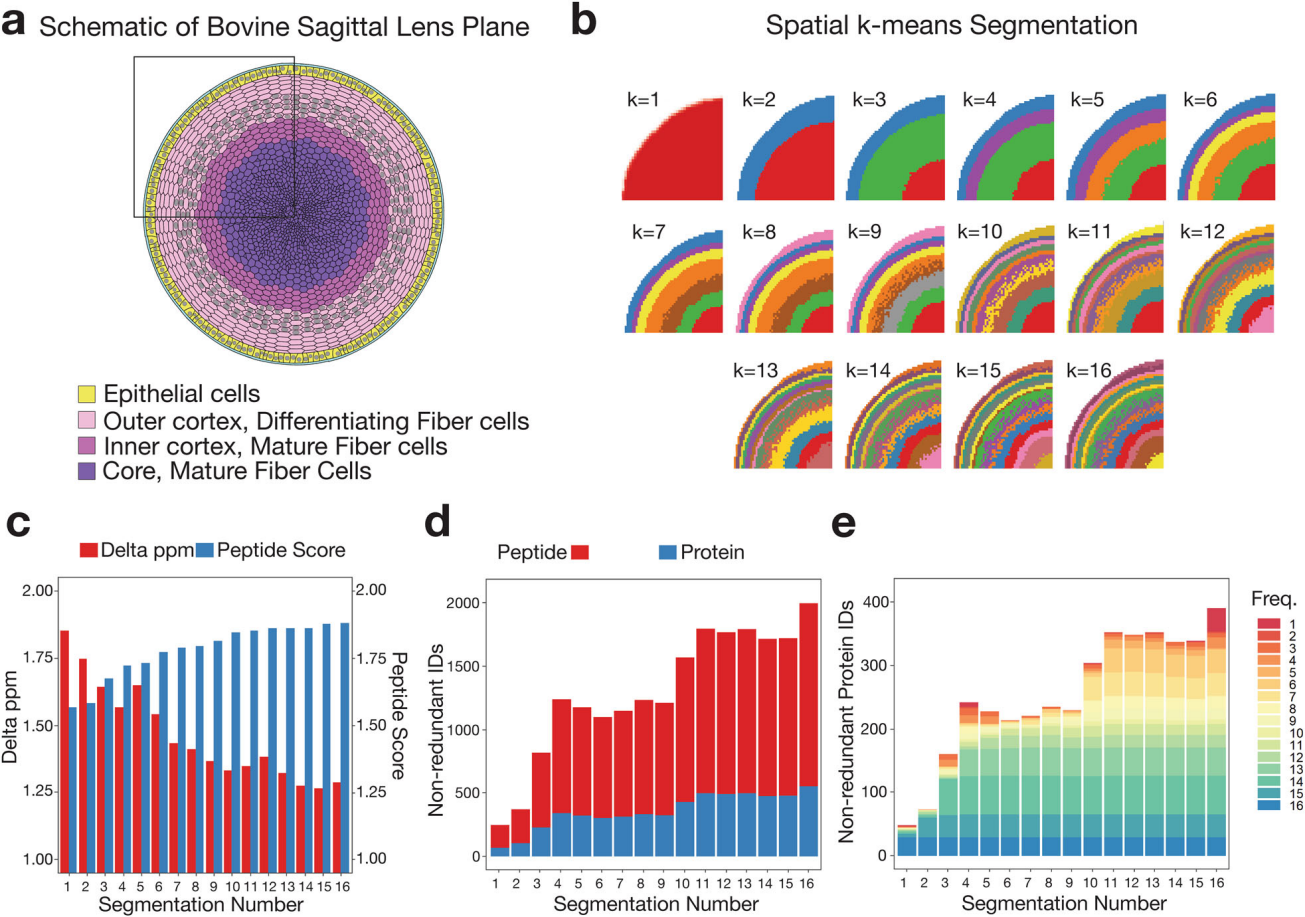
Optimisation of segmentation analysis. **a** Diagrammatic representation of the sagittal plane of the bovine lens detailing the 4 major anatomical regions. **b** HIT-MAP spatial *k*-means clustering of a MALDI–MSI lens dataset based on specification of between *k* = 1 − 16 data-driven segments (colours show individual segments). Note the unbiased annular segmentation in line with lens anatomy. **c** The median mass variance in exact mass-filtering of candidate peptides against *m*/*z* features within the experimental mass spectrum, and the subsequent median peptide score of *m*/*z* features shows improved peptide scoring and declining ppm error during exact mass filtering with increased segmentation. **d** The total number of non-redundant peptide and protein identifications by HIT-MAP with varying segmentation of the lens, and **e** the total number of non-redundant protein identifications by HIT-MAP with varying segmentation of the lens—protein IDs are grouped and coloured by their identification frequency across the serial segmentation test.

Figure 4a, b demonstrates the optimisation of HIT-MAP segmentation steps on the bovine lens dataset. Wang and colleagues^35^ had previously performed MALDI–MSI on a quadrant of a sagittal section of the bovine lens (Fig. 4a) using a Bruker SolariX 15T FT-ICR mass spectrometer. The sample was washed to remove soluble proteins and enrich for structural lens elements prior to imaging. The bovine lens anatomically contains annular segments of developing and mature fibre cells and an outer epithelial layer. The bovine lens dataset was computationally segmented into up to 16 clusters. In line with the underlying biology of lens anatomy, HIT-MAP automatically segmented the MALDI–MSI data in increasing numbers of concentric segments with increasing distance from the lens core (Fig. 4b). We compared a list of performance measures between the differentially segmented lens, to determine the optimal number of segments for HIT-MAP performance. A key consideration to note is that increasing segment number also increases downstream computation time.

Figure 4c shows the mean mass variance in exact mass filtering during PMF analysis, and the subsequent mean peptide score as a function of image segmentation number. We found that while the mass variance typically declines with increased segmentation, the mean peptide score plateaus at around 10 segments for this tissue and there was no significant increase in the number of unique identified proteins above 4 segments (Fig. 4d, e). Of note, segmenting based on 4 anatomical segments provided favourable HIT-MAP performance. The consensus anatomical segmentation for human lens is four, and three for the bovine lens, and our findings confirm that 3–4 segments in computational pixel clustering provided the best performance based on number of robust protein IDs returned with the most efficient use of computational resources. Thus in line with the known anatomy and biology of the lens, we opted for 4 segments in this analysis. HIT-MAP allows users to either manually define segment number, or users can perform an a priori PCA segmentation test to determine the optimal segment number based on protein identifications. Of note is that another drawback of increasing segment number is that smaller segments likely do not contain enough pixels to generate reliable mean spectra for annotation which could increase false positives.

**Fig. 5:**
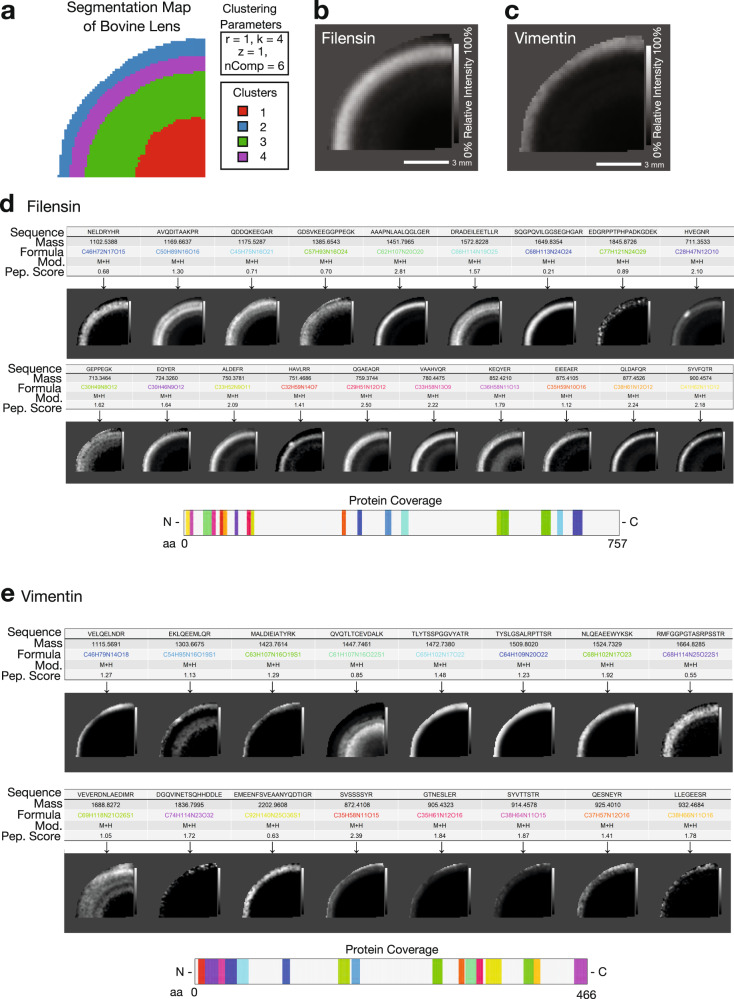
Application of HIT-MAP to a bovine lens FT-ICR mass spectrometry imaging dataset. **a** Spatial *k*-means clustering was performed on the bovine lens into *n* = 4 unsupervised segments based on prior knowledge of lens biology. **b** HIT-MAP protein cluster image of Filensin comprising 19 robustly annotated *m*/*z* features. **c** HIT-MAP protein cluster image of Vimentin comprising 16 *m*/*z* features. **d** Individual peptide distributions and protein coverage for Filensin shown in **b** (scale bar = 3 mm). **e** Individual peptide distributions and protein coverage for Vimentin shown in **c** (scale bar = 3 mm). Intensity scales represent relative intensity from 0 to 100%.

Next, we deployed the complete HIT-MAP pipeline to the bovine lens dataset (Fig. 5 and Supplementary Fig. 2). HIT-MAP parameters were set using an FDR cut-off of 1%, a tolerance of 5 ppm, threshold of 0.5% and *n* = 4 segments (Fig. 5a). HIT-MAP successfully annotated a total of 1087 peptides, of which 940 peptides were non-redundant, resulting in a total of 268 protein annotations passing the scoring criteria. In particular, HIT-MAP annotated 5 of the most common lens-specific proteins with high significance scores including: filensin (Fig. 5b, d), phakinin, α-crystalin (A- and B-chains), and β-crystallin B2 (Supplementary Fig. 2a–e). HIT-MAP also annotated a number of cytoskeletal proteins which based on the current understanding of lens structure and function were expected to be present, including vimentin (Fig. 5c, e), actin-related protein 8, cortactin, arpin (Fig. 2), tropomyosin-4 and α1, myosin light chain-3, dynein regulatory complex subunit 4, and kinesin family member 14. Meanwhile, visinin like protein-1 and ankyrin repeat domain 45 were not expected (based on prior knowledge), suggesting that annotation via HIT-MAP can reveal novel tissue biology (Supplementary Fig. 2f–p).

**Fig. 6:**
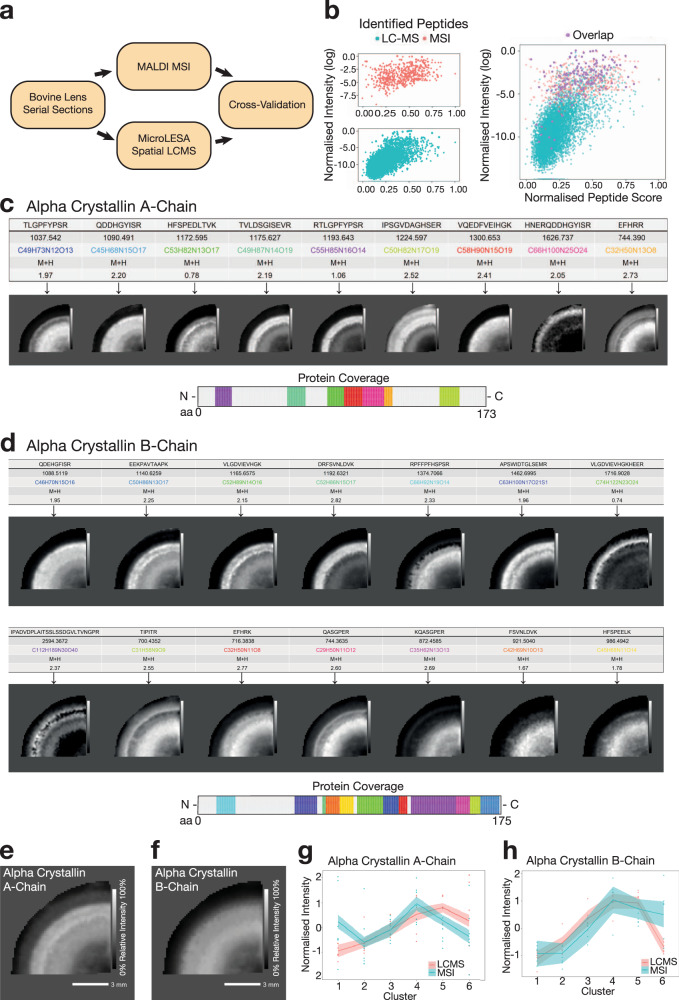
Orthogonal cross-validation of HIT-MAP. **a** HIT-MAP output from the bovine lens dataset was cross-referenced by integrating the bovine lens MSI data with a complementary spatial LC–MS/MS dataset generated from serial sections using liquid micro-enzymatic digest and liquid extraction surface analysis (microLESA). **b** Visualisation of co-annotated peptides from both LC–MS and MSI platforms reveals the greatest degree of overlap occurs in the high-intensity region of the peptide distribution. **c** HIT-MAP annotation of alpha crystallin A-chain showing individual peptide distributions and protein coverage (scale bar = 3 mm). **d** HIT-MAP annotation of alpha crystallin B-chain showing individual peptide distributions and protein coverage (scale bar = 3 mm). **e** HIT-MAP protein cluster image of alpha crystallin A-chain and **f** alpha crystallin B-chain. **g**, **h** Correlation of expression of alpha crystallin A- and B-chains in the microLESA LC–MS regions from^35^ and MALDI–MSI HIT-MAP analysis showing robust overlap of spatial distribution patterns. The light blue/red area shows confidence intervals (95%) of the protein level from two analytical systems, which were estimated using a *t*-based approximation among the observed peptides. Intensity scales represent relative intensity from 0 to 100%.

To validate the HIT-MAP annotations, we cross-referenced our MALDI–MSI data analysis with the previously published spatial LC–MS/MS dataset generated via micro-enzymatic digest and liquid extraction surface analysis (microLESA) from serial sections (Fig. 6a)^35^. MicroLESA sampling of the lens section was originally carried out on 6 predetermined regions of the lens. Therefore, we performed manual segmentation of the lens MALDI–MSI datasets to match cross-referencing to micro-dissected regions of interest (Supplementary Fig. 3a). The manual segmentation function of HIT-MAP allows specific ROI mapping to match the original microLESA regions. Briefly, pixels in the MALDI–MSI image were converted to *x*–*y* coordinates. A manual segmentation configuration file was used to define the matched regions based on the relative distance from the lens centre. Further details on manual segmentation can be found in the README.md.

To account for differences in dynamic range and sensitivity between MALDI–MSI and LC–MS/MS platforms, we performed a relative normalisation of peak intensity and peptide score in order to confidently compare the two datasets (Supplementary Fig. 3b). We found that peptide score is not the most critical factor in determining the degree of overlap between the two platforms (Supplementary Fig. 3b [top right]), while peptide intensity substantially increases the proportion of overlapped peptides (Supplementary Fig. 3b [bottom left]). Using a dual scoring system, integrating relative peptide score and intensity measures allowed a more direct comparison of the two platforms (Supplementary Fig. 3b [bottom right]), yielding a spatial overlap in 87/612 peptides (14.3%) across the 6 ROIs. This overlap is visualised in Fig. 6b, demonstrating mutually annotated peptides in the high-intensity region of the LC–MS/MS dataset.

Importantly, we found a highly statistically significant overlap in 8 annotated proteins between the two platforms, including filensin, phakinin, α-crystallin (A- (Fig. 6c, e) and B- chains (Fig. 6d, f)), β-crystallin B2 and vimentin. Next, we performed a spatial correlation of the two platforms, the MALDI–MSI unbiased segmentation and annotation, and the microLESA captured 6 segments, and found that for the co-annotated lens-specific crystallins as well as vimentin, we see high fidelity and mapping of annotated peptides within segments across the two platforms (Fig. 6g, h and Supplementary Fig. 3c).

**Fig. 7:**
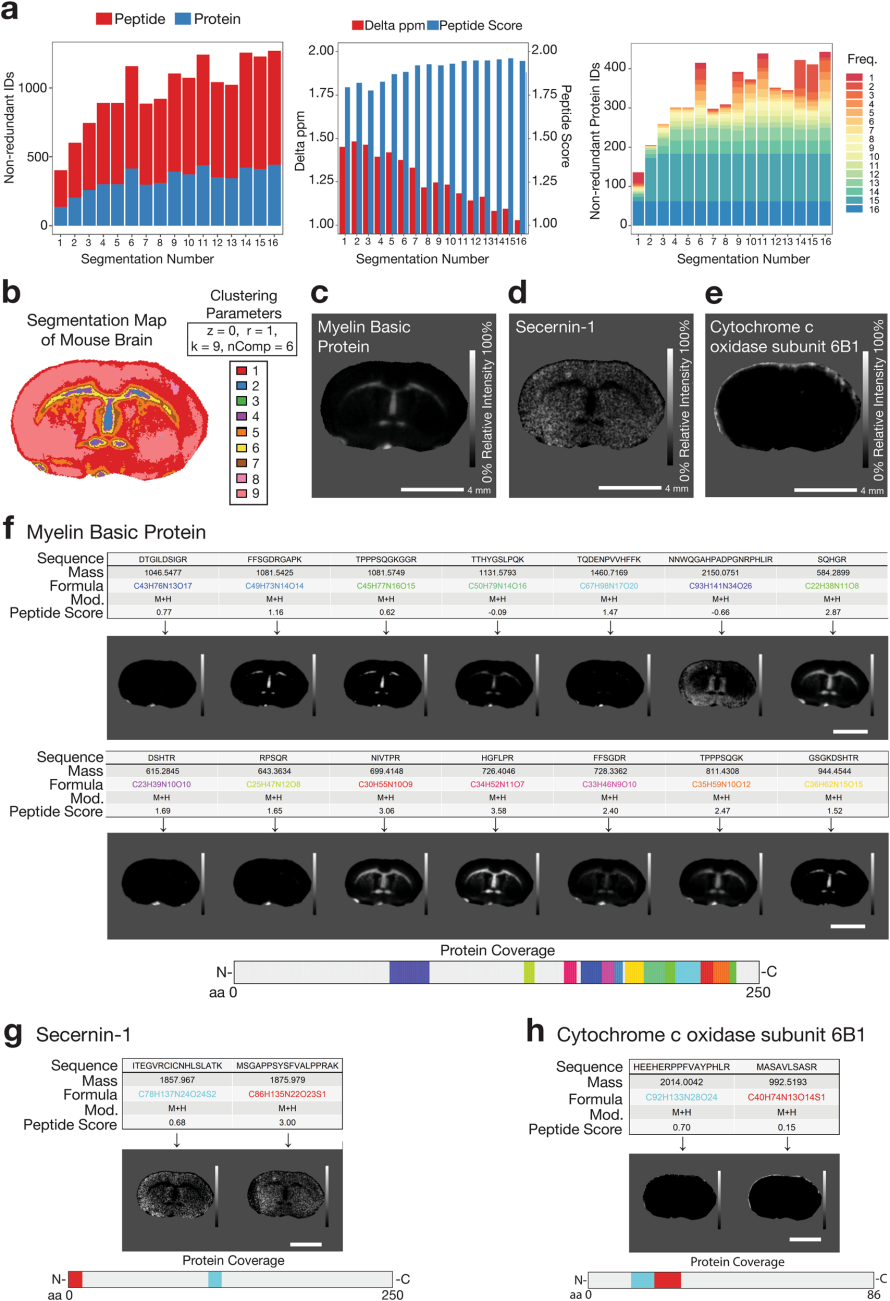
Application of HIT-MAP to a murine brain FT-ICR mass spectrometry imaging dataset. **a** [left] The total number of non-redundant peptide and protein identifications by HIT-MAP with varying segmentation of the brain. [centre] The median mass variance in exact mass-filtering of candidate peptides against *m*/*z* features within the experimental mass spectrum, and the subsequent median peptide score of *m*/*z* features with varying segmentation of the brain. [right] The total number of unique protein identifications by HIT-MAP with varying segmentation of the brain. Protein IDs are grouped and coloured by their identification frequency across the serial segmentation test. **b** Spatial *k*-means segmentation was performed on the brain using *n* = 9 segments. **c** HIT-MAP protein cluster image of Myelin Basic Protein. **d** HIT-MAP protein cluster image of Secernin-1. **e** HIT-MAP protein cluster image of cytochrome *c* oxidase subunit 6B1. **f** Individual peptide distributions and protein coverage for Myelin Basic Protein shown in **c** (scale bar = 4 mm). **g** Individual peptide distributions and protein coverage for Secernin-1 shown in **d**, (scale bar = 4 mm). **h** Individual peptide distributions and protein coverage for cytochrome *c* oxidase subunit 6B1 shown in **e** (scale bar = 4 mm). Intensity scales represent relative intensity from 0 to 100%.

By way of further validation, we performed MALDI–MSI on a coronal (frontal plane) section of the mouse brain to validate the applicability of HIT-MAP in a more complex tissue type with clearly defined organisation (Fig. 7). Rodent brain was selected due to its well-known spatial compartmentalisation of proteins within the grey and white matter regions. In addition, it has been used extensively by the MALDI–MSI community as a model tissue for the development of novel methods, such as detection of membrane^36^ and soluble protein^37,38^, tryptic peptide^39–41^, and endogenous peptide^42,43^ distributions.

MALDI–MSI was performed using a MALDI-FT-ICR mass spectrometer (Bruker SolariX 15T), operated at 50 μm resolution to generate 20,222 spectra with a mass resolving power of 60,000 at *m*/*z* 1046.542. Data were converted to.imzML format and then fed into the HIT-MAP annotation pipeline. Initial HIT-MAP segmentation analysis showed an increase in both peptide and protein identification number as segment number increased (Fig. 7a [left and centre]) similar to that observed in the bovine lens dataset, with stabilisation of protein annotation at 9 segments (Fig. 7a [right]).

The reference in silico database was generated by tryptic digestion of *Mus musculus* proteome (Uniprot FASTA format, accessed 7th January 2021), and the decoy database was generated in isotope mode. HIT-MAP parameters were set using an FDR cut-off of 5%, a tolerance of 5 ppm, a threshold of 0.5%, and *K*-means spatial segmentation was performed with 9 segments.

HIT-MAP robustly annotated 713 peptides correlating to a total of 392 identified unique proteins which showed varying patterns of distribution across the tissue section. In particular, myelin basic protein (Fig. 7c, f) showed exclusive white matter distribution, consistent with its role as a myelinating protein that forms the myelin sheath that facilitates saltatory conduction along neuronal axons. This distribution has been previously reported by two-photon microscopy of optically cleared, whole mouse brain^44^, and also in previous MALDI–MSI work utilising orthogonal tandem MS/MS for identification^39^. Another key example was secernin-1 (Fig. 7d, g) which showed clear localisation in the neuronal cell body-containing grey matter, matching its known localisation as a cytosolic protein, and its role in exocytosis^45^. Of note is that secernin-1 has been reported to accumulate in Alzheimer’s disease plaques^46^. Finally, cytochrome *c* oxidase subunit 6b1 (Fig. 7e, h) also showed a cortical distribution consistent with the localisation of cytochrome oxidase to this brain region using immunohistochemical techniques^47,48^. Additionally, cytochrome *c* oxidase is found in the mitochondrial membrane where it plays an essential role in cellular respiration. Using fluorescent labelling and imaging approaches, Seager and colleagues^49^ have shown localisation in neuronal mitochondria which are known to be sparse in axons (white matter), and abundant in cell soma and dendrites that make up the grey matter, consistent with localisation to the cerebral cortex observed in our MALDI–MSI data.

HIT-MAP also annotated the neuron receptors, GABA receptor γ3, GABA receptor δ, neuronal acetylcholine receptor β3 (Supplementary Fig. 4a–c), as well as synaptic vesicle-associated proteins endophilin-A2, endophilin-B1, syntaxin-1A, vesicle-associated membrane protein 7 and vesicle-associated membrane protein 8 (Supplementary Fig. 4d–h) and signalling proteins protein kinase c eta type and smad nuclear interacting protein-1 (Supplementary Fig. 4i, j). In addition to these, HIT-MAP annotated the metabolic proteins cytochrome *c* oxidase subunit 6A1 and pyruvate kinase (Supplementary Fig. 4k, l); the Alzheimer’s disease-associated proteins neutrophilic granule protein and small EDRK rich factor 2 (Supplementary Fig. 4m, n); and the extracellular proteins a disintegrin and metalloproteinase domain-containing protein 22 (ADAM-22), hyaluronan and proteoglycan link protein 3, and wnt-2b (Supplementary Fig. 4o–q).

Together these data support the accurate annotation of peptides and proteins in the complex tissue of the brain and validate HIT-MAP as a robust platform for the interrogation of MSI datasets.

## Discussion

This study presents the development and application of an open-source computational pipeline for automated identification, annotation and image generation of spatial peptide MALDI mass spectrometry imaging datasets where corresponding orthogonal LC–MS data are not available. The source code for HIT-MAP is freely available from Github [https://github.com/MASHUOA/HiTMaP/].

The development and deployment of the HIT-MAP pipeline are demonstrated through analysing a known standard peptide calibrant mixture, before applying it to two independent real-world biological sample datasets. As a proof of principle, we use both a normal bovine lens, and murine brain dataset, although HIT-MAP could be applied to any normal or disease context. The annotations and corresponding peptide and protein MALDI images generated by HIT-MAP have been validated by interrogation of bovine lens peptide identities determined by orthogonal LC–MS/MS analysis, while brain annotations were cross-referenced to literature.

HIT-MAP uses common MALDI–MSI file formats in an executable proteomic workflow for peptide and protein annotation via an FDR-controlled segmentation-based PMF and protein coverage analysis pipeline. A PMF approach has been used for several reasons. Firstly, while there are examples of combined MS and MS/MS MALDI–MSI analysis^50^, MALDI–MSI instrumentation generally acquires in either MS or MS/MS mode because of the pulsatile generation and transmission of ions via MALDI, the destructive nature of MALDI sampling, and the relatively low scan speed in the case of FT-ICR mass analysis. The MS spectra, therefore, contain a spatially resolved proteome, where validating the spatial distribution of a single peptide by comparing it to peptides from the same protein, i.e. a PMF, is useful. The gold standard identification for peptides is via an MS/MS approach to generate peptide fragmentation spectra. While concomitant MS/MS MALDI–MSI sampling is possible, the experimental and instrument time cost to run multiple MS/MS MALDI–MSI is considerable when tens to hundreds of peptides are detected in a single MS MALDI–MSI experiment. Additionally, this may not always be possible since it generally requires a serial section approach, and tissue availability may be limiting in prospective analysis and not possible in retrospective analysis. A key strength of HIT-MAP is the ability to retrospectively re-analyse already generated MALDI–MSI datasets already in the community, as well as its potential application to archived Formalin Fixed Paraffin Embedded (FFPE) tissue sections in an unbiased manner, providing versatility in exploratory analysis over multiplex immunohistochemistry approaches.

Finally, MS/MS analysis requires sufficient ion intensity to acquire high-quality peptide fragmentation spectra. Therefore, low abundance peptides are unlikely to contribute to positive identification of a protein distribution in MS/MS mode, but collectively can provide validation of protein spatial distribution using a PMF approach. While HIT-MAP can correctly annotate and plot peptide and protein distributions using this PMF approach, on-tissue MS/MS, even in non-MSI mode, should be performed where possible as an additional step to validate peptide identity.

Validation of spatial distribution and identification of HIT-MAP annotations highlights how it could be used to provide valuable insight into the underlying biology of the analysed tissue(s). Using our ocular lens example, the observed distributions and identifications are consistent with known lens structure and biology. The lens consists of highly elongated fibre cells that are retained throughout life and are spatially organised such that the lens centre is populated by cells that are formed in utero, while new, young fiber cells are localised to the lens edge and continuously added throughout life. Due to this intrinsic cell age gradient, and the cell differentiation events that lens cells undergo as they age, such as degradation of cell nuclei and endoplasmic reticulum, protein distributions, and protein post-translational modifications, are known to change in different lens regions. These changes are linked to functional changes in different lens regions, that allow the whole lens to perform its primary function to focus light on the retina. Consequently, characteristic concentric rings of protein localisation are formed, and have been shown for soluble crystallin proteins by many techniques, including MALDI–MSI^51,52^, as well as membrane proteins^53^.

In the current data, the lens tissue sections were washed extensively prior to matrix application to prepare them for intermediate filament peptide and protein detection. Therefore the predominance of these proteins (i.e. filensin, phakinin, vimentin), correctly annotated by HIT-MAP and localised in concentric rings (Fig. 4), is consistent with both the known tissue preparation, and existing, recently published lens biology^35^. Moreover, the annotation and spatial distribution of arpin (Fig. 2), a cytoskeleton-associated actin-regulating protein, suggests that this protein may be involved in the marked changes to the lens fibre cell cytoskeleton that are proposed to take place during lens cell differentiation and aging^54^.

We do note that occasionally some peptides assigned to a particular protein show a different distribution to the majority of other peptides from that protein (see e.g. Figs. 5 and 7f). This could be due to several reasons. Computationally, this could be due to the overlap of two or more peptide isotopic envelopes from different regions. Experimentally, the suppression of a *m*/*z* feature in different tissue regions may explain spatial discordance. Alternatively, this could be due to interpretable biology. For example, lens crystallin proteins undergo extensive post-translational truncation, such that the origin of a tryptic peptide could be from either the full-length or truncated version. There may therefore be spatial differences between different tryptic peptides from the different crystallin protein forms, despite their correct assignment. Since MALDI–MSI offers the potential for unbiased exploration of protein distribution, strict spatial filtering of assigned peptides could potentially lead to inaccurate biological interpretation. Therefore, we have designed HIT-MAP to initially include and plot all matched peptides that pass the under-defined, FDR-controlled scoring system irrespective of their spatial distribution, while simultaneously providing the calculated peptide score in the results table for researchers to assess. HIT-MAP users should be aware of the potential discordance of a small number of annotated peptides, however, HIT-MAP plots a protein-level spatial distribution image which is based on weighted scoring of all peptides assigned to protein identification. For example, in the bovine lens model presented (Fig. 5), the predominance of the signal is localised to the lens periphery and cortex as has been shown by previous orthogonal approaches in the field, while in the brain, myelin basic protein distribution is predominantly in white matter, consistent with previous MSI studies^39,40^.

Together, our results demonstrate the potential utility of HIT-MAP in the analysis of enzymatically generated peptide MALDI-MSI spatial mapping studies of biological tissues. Importantly, HIT-MAP allows researchers to overcome one of the biggest technical limitations associated with determining peptide identity. At present, spatial identification and quantification of peptides are mutually exclusive during MALDI–MSI acquisition. Post-acquisition manual analysis of spectra is time-consuming and often results in publications reporting feature *m*/*z* values alone without identification. Alternatively, it requires the additional generation of spectral datasets from orthogonal databases generated via LC–MS/MS on matched tissues which may not be possible where the material is limited, such as in patient biopsies. Furthermore, HIT-MAP operates independently of the acquisition platform allowing researchers to revisit and re-analyse retrospective mass spectrometry imaging datasets to draw new information.

HIT-MAP addresses a current area of unmet need in the mass spectrometry imaging field, offering a platform-independent, open-source pipeline for the automated annotation and visualisation of high-resolution proteomic MALDI–MSI datasets, both newly generated and retrospective, which will be of significant value to the mass spectrometry imaging community. Furthermore, the ability to integrate MALDI–MSI with other established (i.e. routine histology) and emerging genome-centric technology platforms such as spatial transcriptomics, using multi-layered co-registration approaches will greatly increase the depth of knowledge generated in understanding functional gene-to-protein expression patterns in both healthy and diseased contexts.

## Methods

### MALDI–MSI sample preparation

For validation experiments using peptide standards, one microliter of Bruker peptide calibrant II (prepared as per manufacturer’s instructions) was spotted onto an Indium tin oxide (ITO)-coated glass slide. After air-drying, the slide was coated with α-Cyano-4-hydroxycinnamic acid (CHCA, 7 mg/mL) in 50%ACN/0.1% TFA using a TM-sprayer (HTX Technologies, NC, USA). The sprayer settings were 700 mm/min velocity, 2 mm track spacing, 8 passes, 0.1 mL/min flow rate. Following air-drying, the sample was stored in a vacuum desiccator until MALDI–MSI analysis.

Both the bovine lens and mouse brain tissue were purchased from Pel-Freez Biologicals (Rogers, AR). For tissue imaging sample preparation, full sample preparation information for the bovine lens data is available in ref. ^35^. Mouse brain was sectioned to 10 µm thickness. The section was washed sequentially as follows: 50 mM ammonium formate wash for 1 min twice, dried, water wash for 1 min, dried, Carnoy’s solution wash for 2 min, dried and 95% ethanol wash for 2 min, dried. On-tissue digestion was performed using trypsin (15 ng/µL) applied in 10% ACN in 100 mM ammonium bicarbonate, pH 8 using a TM-Sprayer (HTX Technologies, Carrboro, NC, USA) modified with a syringe pump at 8 µL/min (Harvard Apparatus, Holliston, MA, USA). Trypsin was applied in eight passes with a nozzle velocity of 750 mm/min at 30 °C. The final trypsin concentration on tissue was 0.64 ng/mm^2^. Digestion was done in a sealed humidified petri dish at 37 °C with 0.1 mL 100 mM ammonium bicarbonate overnight (16–18 h)^35^. After air-drying, the slide was coated with α-cyano-4-hydroxycinnamic acid (CHCA, 5 mg/mL) in 90%ACN/0.1% TFA for bovine lens sections or 2,5-dihydroxybenzoic acid (15 mg/mL DHB) for mouse brain sections using a TM-sprayer (HTX Technologies, NC, USA). The sprayer settings were 700 mm/min velocity, 2 mm track spacing, 8 passes, 0.1 mL/min flow rate. Following air-drying, the sample was stored in a vacuum desiccator until MALDI–MSI analysis.

### MALDI–MSI image acquisition

Peptide calibrant spot imaging data were acquired using a SolariX XR 7T FT-ICR mass spectrometer equipped with a dual MALDI/ESI source and a dynamically harmonised ParaCell (Bruker Daltonics, Billerica, MA) and operated using ftmsControl v.2.2. The MALDI source employs a Smartbeam II Nd:YAG laser system (2 kHz, 355 nm). Data were collected in positive ion mode from *m*/*z* 150−5000 with a resolving power (*m*/Δ*m*) of 99,000 at *m*/*z* 400. Tandem mass spectrometry was performed using an isolation window of 3 or 10 Da to maximise signal without interference from other ions, and the collision energy was set to 40 V.

Tissue imaging experiments were performed using a Bruker SolariX 15T FT-ICR mass spectrometer (Bruker Daltonics, Billerica, MA, USA) equipped with a dual MALDI/ESI source and a dynamically harmonised ParaCell (Bruker Daltonics, Billerica, MA) and operated using ftmsControl v.2.2. The MALDI source employs a Smartbeam II Nd:YAG laser system (2 kHz, 355 nm). Data were collected in positive ion mode from *m*/*z* 500–3000 with a resolving power (*m*/Δ*m*) of 80,000 at *m*/*z* 1046.542 for bovine lens data and *m*/*z* 500–3000 with a resolving power of 60,000 for mouse brain data. A raster step size of 150 μm was used for lens data and 50 µm was used for brain data.

### Reporting summary

Further information on research design is available in the Nature Research Reporting Summary linked to this article.

## Supplementary information

Supplementary Information

Reporting Summary

## Data Availability

Mass spectrometry data pertaining to the Bruker peptide calibrant are available from the corresponding authors upon reasonable request. The data pertaining to the lens bovine tissue and mouse brain tissue mass spectrometry imaging datasets have been deposited to the ProteomeXchange Consortium via the PRIDE partner repository^55^ with the dataset identifier PXD025486. All other relevant data supporting the findings are available within the paper and its Supplementary Information files.

## Code availability

HIT-MAP is open source and freely available for download from Github at https://github.com/MASHUOA/HiTMaP alongside user documentation. HIT-MAP is also available as a Docker Image at https://hub.docker.com/r/mashuoa/hitmap. The README file contains additional information on all of the available parameters of HIT-MAP, including examples of use. Developed using R version 3.6 or above, HIT-MAP is released as a standard R package. The whole package was built on R using RStudio. Cardinal^23^ has been used for .imzML data pre-possessing, image segmentation and ion image plotting. Cleaver^26^ and Biostrings^25^ were used for peptide candidate generation and protein coverage calculation. protViz^27^ was used to translate peptide sequence into formula and calculate parent mass. rcdk^29^ and rcdklibs^30^ were used for isotopic pattern generation. FTICRMS^56^ and OrgMassSpecR^57^ was used for high-resolution spectrum processing and isotopic peak similarity scoring. BiocParallel^21^ was recruited to perform parallel processing on different operating systems. colortools^58^ was used to generate a colour set for a given number of components. Magick^59^ was used for output image handling. Other fundamental generic packages in R were also used to build the HIT-MAP package including knitr^60^, dbplyr^61^, xml2^62^, stringr^63^, ggplot2^64^, reticulate^65^, rJava^66^, ncdf4^67^, tibble^68^, purrr^69^ and rcpp^70^.
