# Supplementary material for: Automated annotation and visualisation of high-resolution spatial proteomic mass spectrometry imaging data using HIT-MAP
Source: Nat Commun. 2021 May 28;12:3241. doi: 10.1038/s41467-021-23461-w (PMC8163805; doi:10.1038/s41467-021-23461-w)
Supplement: Supplementary file 1 — Supplementary Information [file 41467_2021_23461_MOESM1_ESM.pdf]

Supplementary Information

Automated annotation and visualisation of high-resolution spatial proteomic mass spectrometry imaging data using HIT-MAP

Guo *et al.*

Supplementary Figure 1

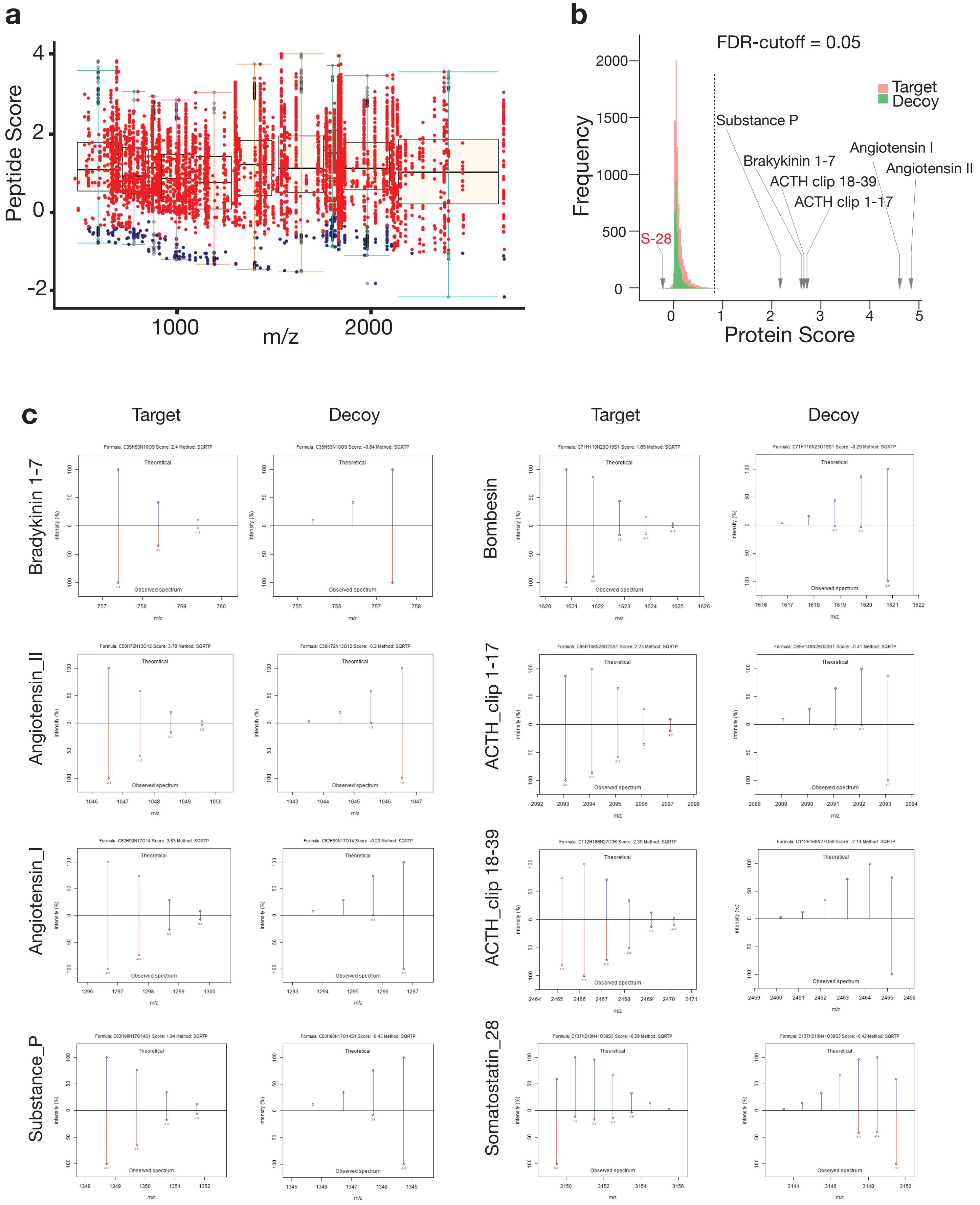

Supplementary Figure 1 cont.

d

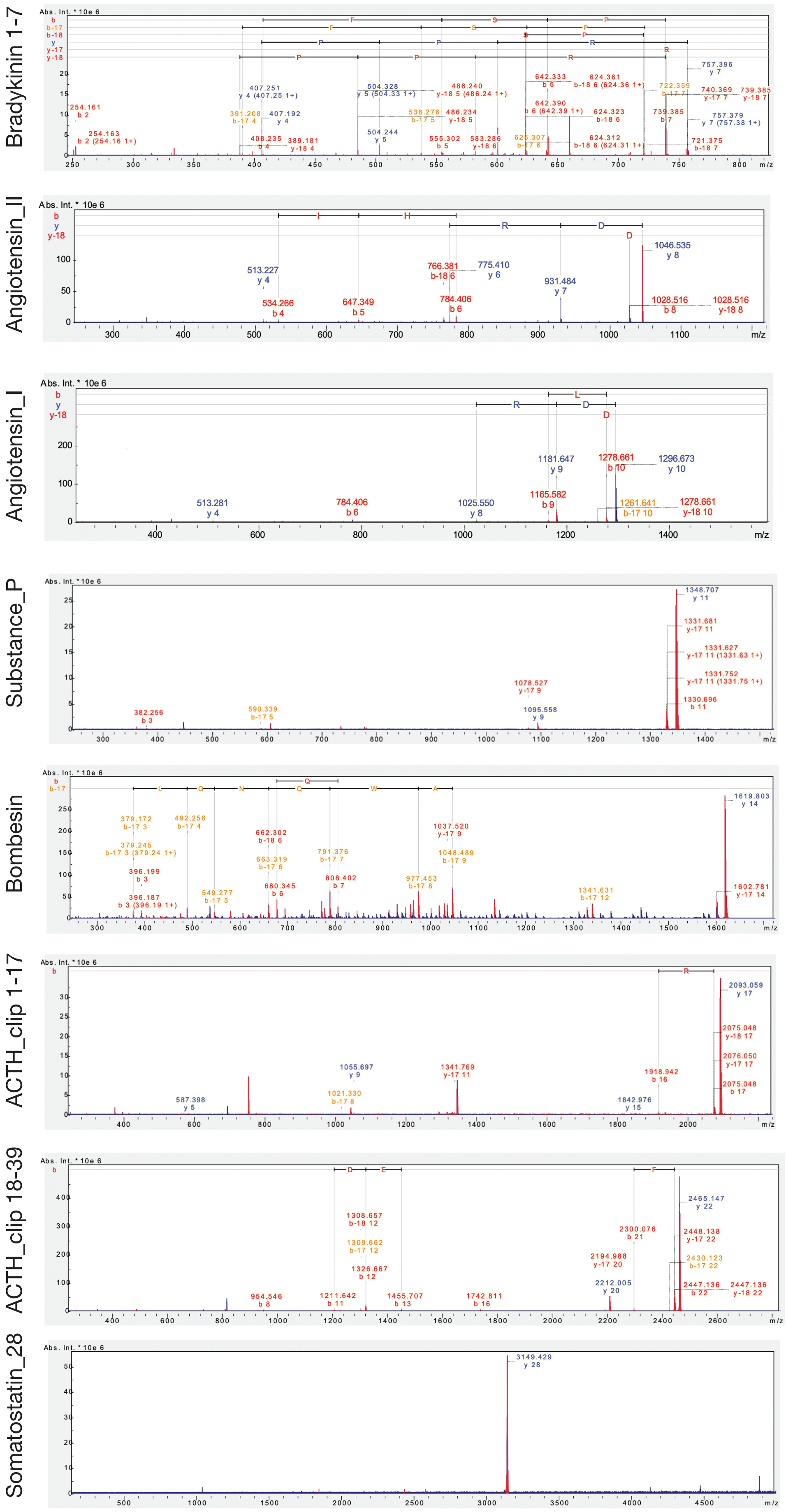

Supplementary Figure 1 | **a.** Post-hoc IQR outlier analysis of peptide score across the mass ranges identified in the peptide calibrant identifies putative outlier peptides (blue dots) using an interquartile-range (IQR) function (box plots how minimum, maximum and interquartile range). **b.** The frequency distribution of protein candidate list scoring for the preliminary HIT-MAP analysis, annotating 7/8 proteins (excluding Somatostatin-28). **c.** The peptide mass fingerprint analysis demonstrating cross-referencing of the observed isotopic pattern to the simulated theoretical target or decoy isotopic pattern for the 8 peptide calibrants. **d.** MALDI-FT-ICR-MS/MS of the 8 peptides present with the calibrant.

Supplementary Figure 2

a Filensin

NELDRYHR	AVQDITAAKPR	QDDQKEEGAR	GDSVKEEGGPPEGK	AAAPNLAALQGLGER	DRADEILEETLLR	SQGPQVILGGSEGHGAR	EDGRPPTPHPADKGDEK	HVEGNR	GEPPEGK
1102.5388	1169.6637	1175.5287	1385.6543	1451.7965	1572.8228	1649.8354	1845.8726	711.3533	713.3464
$C_{46}H_{72}N_{17}O_{15}$	$C_{50}H_{89}N_{16}O_{16}$	$C_{45}H_{75}N_{16}O_{21}$	$C_{57}H_{93}N_{16}O_{24}$	$C_{62}H_{107}N_{20}O_{20}$	$C_{66}H_{114}N_{19}O_{25}$	$C_{68}H_{113}N_{24}O_{24}$	$C_{77}H_{121}N_{24}O_{29}$	$C_{28}H_{47}N_{12}O_{10}$	$C_{30}H_{49}N_{8}O_{12}$
M+H	M+H	M+H	M+H	M+H	M+H	M+H	M+H	M+H	M+H
0.68	1.30	0.71	0.70	2.81	1.57	0.21	0.89	2.10	1.62

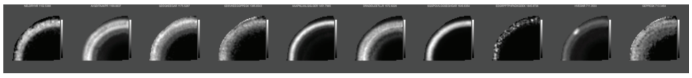

EQYER	ALDEFR	HAVLRR	QGAEAQR	VAAHVQR	KEQYER	EIEEAER	QLDAFQR	SYVFQTR
724.3260	750.3781	751.4686	759.3744	780.4475	852.4210	875.4105	877.4526	900.4574
$C_{30}H_{46}N_{9}O_{12}$	$C_{33}H_{52}N_{9}O_{11}$	$C_{32}H_{59}N_{14}O_{7}$	$C_{29}H_{51}N_{12}O_{12}$	$C_{33}H_{58}N_{13}O_{9}$	$C_{36}H_{58}N_{11}O_{13}$	$C_{35}H_{59}N_{10}O_{16}$	$C_{38}H_{61}N_{12}O_{12}$	$C_{41}H_{62}N_{11}O_{12}$
M+H	M+H	M+H	M+H	M+H	M+H	M+H	M+H	M+H
1.64	2.09	1.41	2.50	2.22	1.79	1.12	2.24	2.18

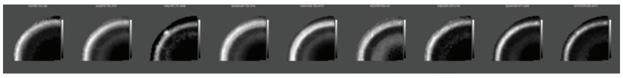

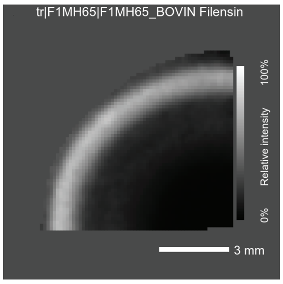

b Phakinin

LQAGALLQAK	YENEQPFR	VSQELEAQLR	YENEQPFRK	DVASYHALLDR	ATRSENWGALR	SSGLATAPAPSLER	AAQSPSSLDGLPASR	VYVGMAPSGPTGGLGAR
1012.6150	1082.4902	1172.6270	1210.5851	1259.6379	1260.6444	1356.7118	1456.7390	1589.8104
$C_{45}H_{82}N_{13}O_{13}$	$C_{48}H_{68}N_{13}O_{16}$	$C_{49}H_{86}N_{15}O_{18}$	$C_{54}H_{80}N_{15}O_{17}$	$C_{55}H_{87}N_{16}O_{18}$	$C_{53}H_{86}N_{19}O_{17}$	$C_{57}H_{98}N_{17}O_{21}$	$C_{60}H_{102}N_{19}O_{23}$	$C_{69}H_{113}N_{20}O_{21}S_{1}$
M+H	M+H	M+H	M+H	M+H	M+H	M+H	M+H	M+H
0.28	0.93	-0.36	1.09	1.62	0.53	2.39	1.39	1.20

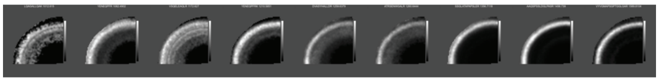

VVVDAPAGASSSMPLQR	DVASYHALLDREESS	RVVVDAPAGASSSMPLQR	IHWER	MHLESK	QQAELAR	TVAVSGLVR	SENWGALR
1684.8687	1691.7871	1840.9698	740.3838	744.3709	815.4370	901.5465	932.4585
$C_{71}H_{122}N_{21}O_{24}S_{1}$	$C_{71}H_{111}N_{20}O_{28}$	$C_{77}H_{134}N_{25}O_{25}S_{1}$	$C_{34}H_{50}N_{11}O_{8}$	$C_{31}H_{54}N_{9}O_{10}S_{1}$	$C_{33}H_{59}N_{12}O_{12}$	$C_{39}H_{73}N_{12}O_{12}$	$C_{40}H_{62}N_{13}O_{13}$
M+H	M+H	M+H	M+H	M+H	M+H	M+H	M+H
1.22	0.68	1.72	2.39	1.94	2.64	1.79	2.20

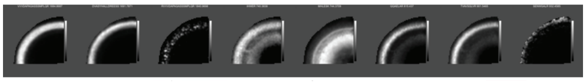

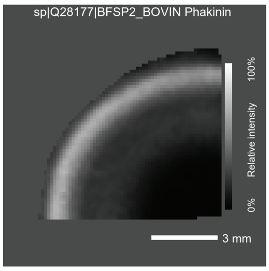

c Alpha Crystallin A Chain

TLGPFYPSR	QDDHGYISR	HFSPEDLTVK	TVLDSGISEVR	RTLGPFYPSR
1037.542	1090.491	1172.595	1175.627	1193.643
$C_{49}H_{73}N_{12}O_{13}$	$C_{45}H_{68}N_{15}O_{17}$	$C_{53}H_{82}N_{13}O_{17}$	$C_{49}H_{87}N_{14}O_{19}$	$C_{55}H_{85}N_{16}O_{14}$
M+H	M+H	M+H	M+H	M+H
1.97	2.20	0.78	2.19	1.06

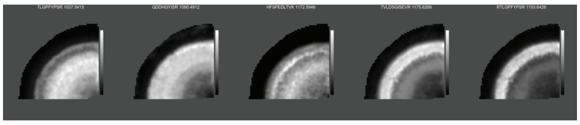

IPSGVDAGHSER	VQEDFVEIHGK	HNERQDDHGYISR	EFHRR
1224.597	1300.653	1626.737	744.390
$C_{50}H_{82}N_{17}O_{19}$	$C_{58}H_{90}N_{15}O_{19}$	$C_{66}H_{100}N_{25}O_{24}$	$C_{32}H_{50}N_{13}O_{8}$
M+H	M+H	M+H	M+H
2.52	2.41	2.05	2.73

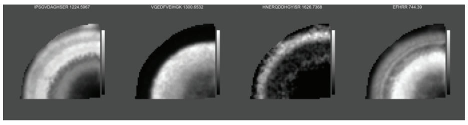

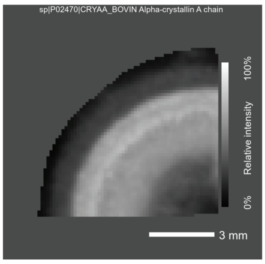

d Alpha Crystallin B Chain

QDEHGFISR	EEKPAVTAAPK	VLGDVIEVHGK	DRFSVNLDVK	RPFFPFHSPSR	APSWIDTGLSEMR	VLGDVIEVHGKHEER
1088.5119	1140.6259	1165.6575	1192.6321	1374.7066	1462.6995	1716.9028
$C_{46}H_{70}N_{15}O_{16}$	$C_{50}H_{86}N_{13}O_{17}$	$C_{52}H_{89}N_{14}O_{16}$	$C_{52}H_{86}N_{15}O_{17}$	$C_{66}H_{92}N_{19}O_{14}$	$C_{63}H_{100}N_{17}O_{21}S_{1}$	$C_{74}H_{122}N_{23}O_{24}$
M+H	M+H	M+H	M+H	M+H	M+H	M+H
1.95	2.25	2.15	2.82	2.33	1.96	0.74

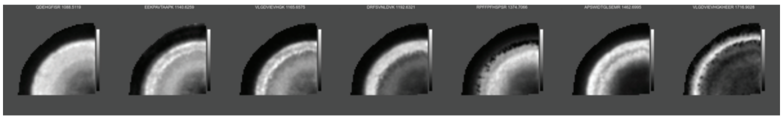

IPADVDPLAITSSLSSDGVLTVNGPR	TIPITR	EFHRK	QASGPER	KQASGPER	FSVNLDVK	HFSPEELK
2594.3672	700.4352	716.3838	744.3635	872.4585	921.5040	986.4942
$C_{112}H_{189}N_{30}O_{40}$	$C_{31}H_{58}N_{9}O_{9}$	$C_{32}H_{50}N_{11}O_{8}$	$C_{29}H_{50}N_{11}O_{12}$	$C_{35}H_{62}N_{13}O_{13}$	$C_{42}H_{69}N_{10}O_{13}$	$C_{45}H_{68}N_{11}O_{14}$
M+H	M+H	M+H	M+H	M+H	M+H	M+H
2.37	2.55	2.77	2.60	2.69	1.67	1.78

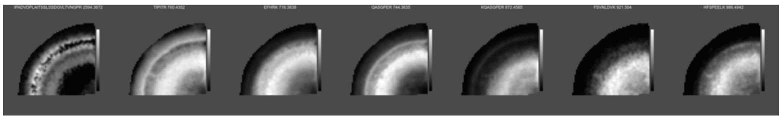

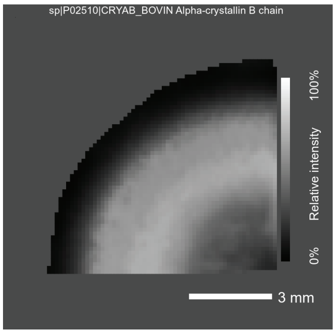

e Beta Crystalin B2

DMQWHQR	TDSLSSLRPIK	IRDMQWHQR	RTDSLSSLRPIK	ITLYENPNFTGK	VDSQEHKITLYENPNFTGK	GAFHPSS	VDSQEHK
1000.4418	1216.6896	1269.6269	1372.7907	1396.7107	2220.0931	702.3206	842.4003
$C_{42}H_{62}N_{15}O_{12}S_{1}$	$C_{52}H_{94}N_{15}O_{18}$	$C_{54}H_{85}N_{20}O_{14}S_{1}$	$C_{58}H_{106}N_{19}O_{19}$	$C_{64}H_{98}N_{15}O_{20}$	$C_{98}H_{151}N_{26}O_{33}$	$C_{31}H_{44}N_{9}O_{10}$	$C_{34}H_{56}N_{11}O_{14}$
M+H	M+H	M+H	M+H	M+H	M+H	M+H	M+H
0.94	1.68	0.92	0.64	0.53	2.45	3.01	0.96

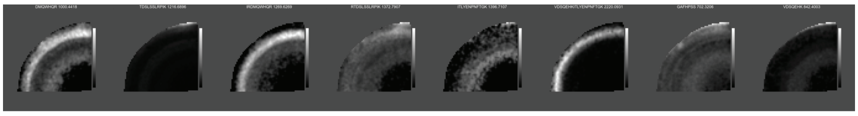

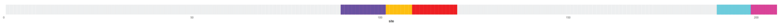

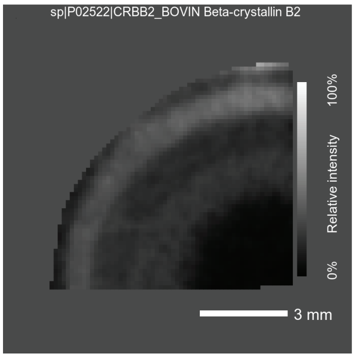

Supplementary Figure 2 cont.

f Vimetin

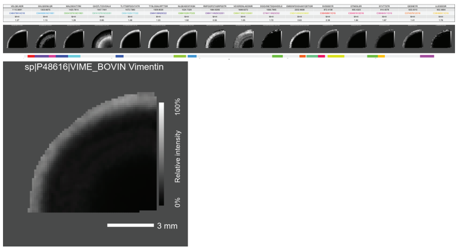

g Actin Related Protein 8

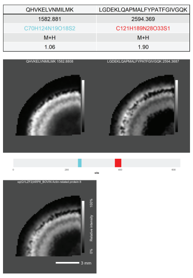

h Visinin Like Protein 1

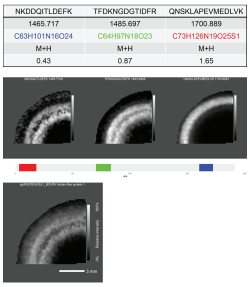

i Cortactin

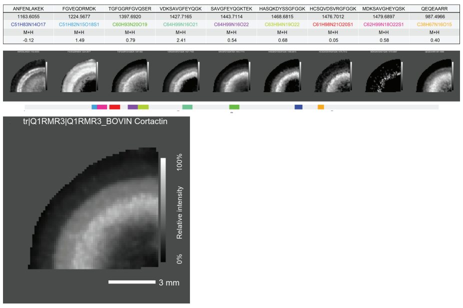

j Arpin

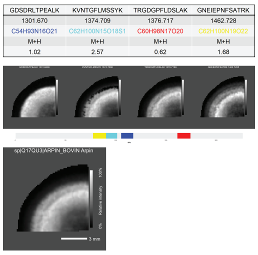

k Tropomyosin 4

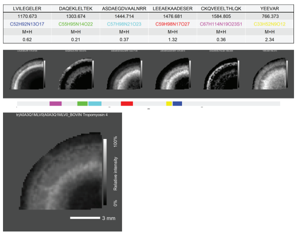

l Tropomyosin Alpha 1

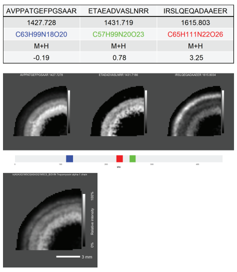

m Myosin Light Chain 3

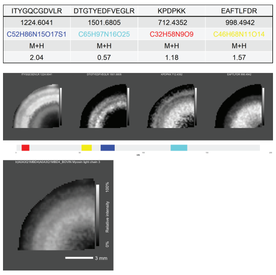

n Dynein Regulatory Complex Subunit 4

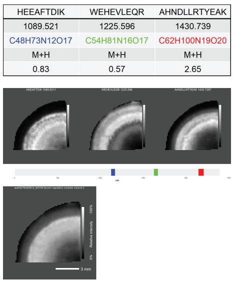

o Kinesin Family Member 14

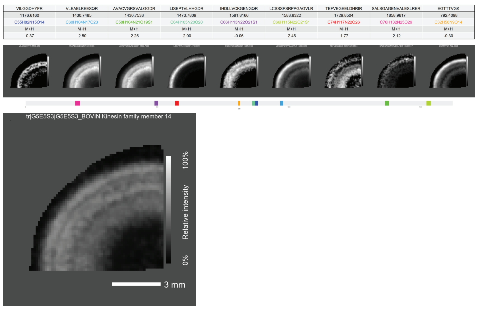

p Ankyrin Repeat Domain 45

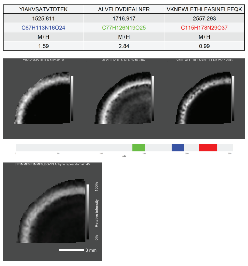

Supplementary Figure 2 | HIT-MAP analysis of published bovine lens(Wang et al. 2020). All peptide and protein cluster imaging outputs of annotated **a-e.** lens and **f-p.** cytoskeletal proteins, (scale bar = 3mm). Intensity scales represent relative intensity from 0% to 100%.

Supplementary Figure 3

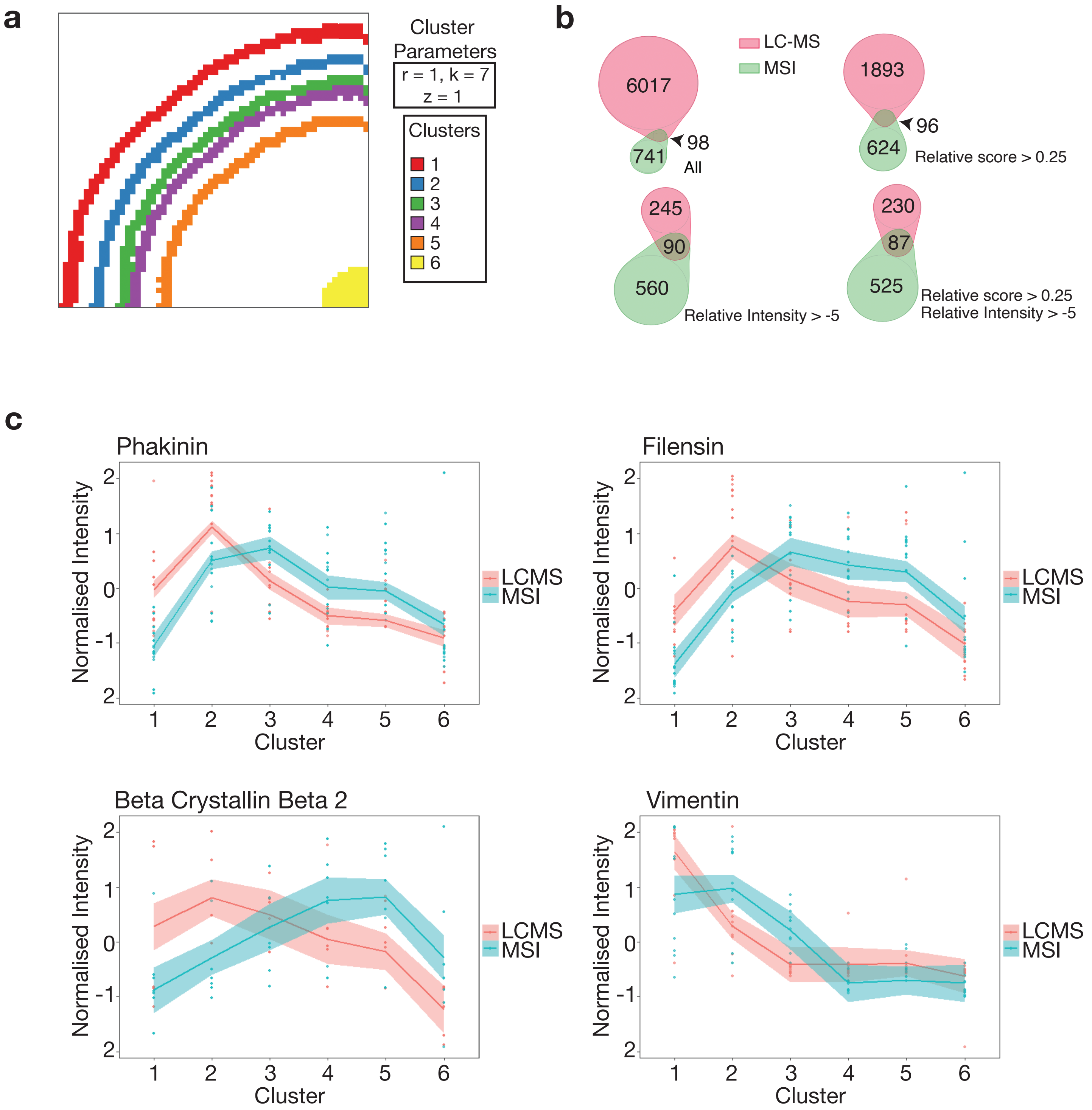

Supplementary Figure 3 | **a.** Segmentation of published bovine lens(Wang et al. 2020) for cross-referencing to spatial microLESA captured regions that underwent LC-MS/MS on a serially sectioned bovine lens MALDI dataset. **b.** Cross-referencing of the annotated peptides from the MALDI-MSI HIT-MAP pipeline with the LC-MS dataset. The relative overlap in co-annotated peptides increases when implementing either a relative score (ii), and relative intensity (iii) or both (iv), as compared to no normalisation (i). **c.** Spatial correlation of additional LC-MS/MS validated proteins annotated by the HIT-MAP pipeline. Intensity scales represent relative intensity from 0% to 100%.

Supplementary Figure 4

a GABA receptor γ3 subunit

TTLGITTVLTMTTLSTIAR	SGSWRR
1994.1202	748.3849
C86H157N22O29S1	C31H50N13O9
M+H	M+H
2.72	1.87

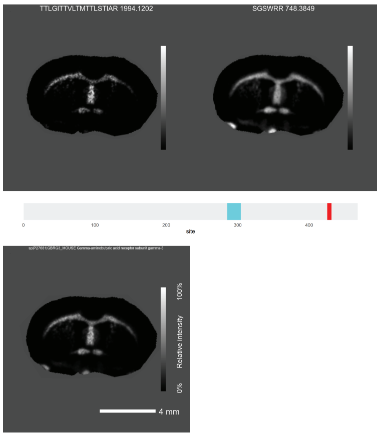

b GABA receptor δ subunit

EGGSRPGGPGGIR	VSLGITTVLTMTTLMVSAR
1196.613	1994.102
C48H82N19O17	C86H157N22O27S2
M+H	M+H
-0.10	1.89

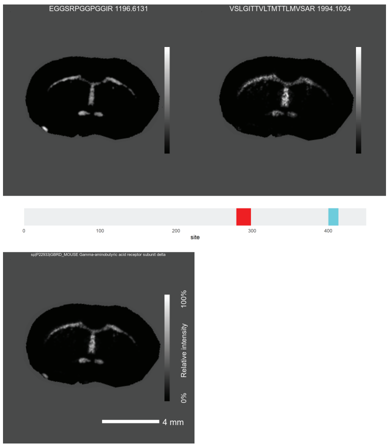

c Neuronal acetylcholine receptor β3 subunit

LPRWLCMK	SSSTYHPMAPWVKR	VYFGLK
1046.5638	1646.8108	726.4185
C48H80N13O9S2	C74H112N21O20S1	C37H56N7O8
M+H	M+H	M+H
1.12	-0.59	1.64

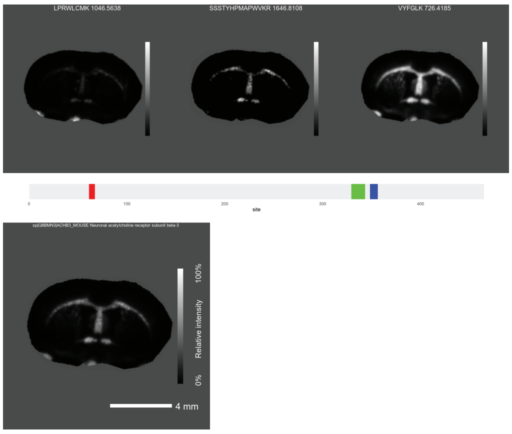

d Endophilin-A2

QGKIPDEELR	MPSKSMPPLDQPSCK	ITASSSFR
1184.6270	1645.7746	868.4523
C50H86N15O18	C69H117N18O22S3	C37H62N11O13
M+H	M+H	M+H
0.81	-0.02	1.96

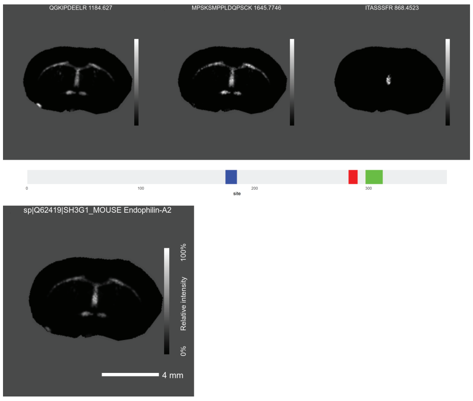

e Endophilin-B1

AAETKSSSEQELR	LGQAEKTELDAHLENLLSK
1435.702	2109.119
C57H99N18O25	C91H154N25O32
M+H	M+H
0.92	2.77

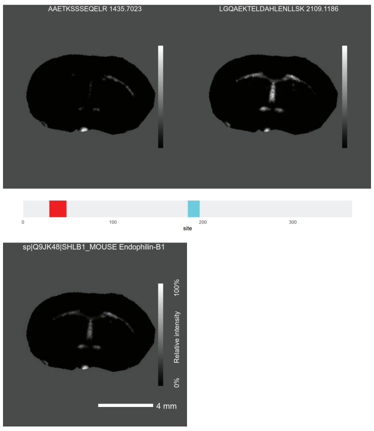

f Syntaxin-1A

EELEELMSDIK	HSEIIK
1335.6348	726.4145
C56H95N12O23S1	C32H56N9O10
M+H	M+H
-1.31	3.82

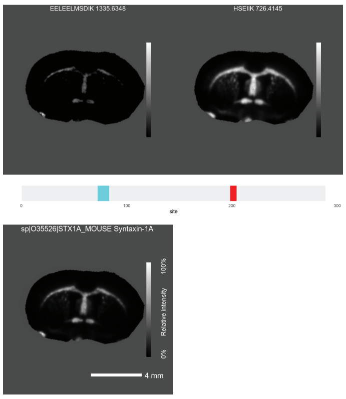

g Vesicle-associated membrane protein 7

LELLIDKTENLVDSSVTFK	AMCMK
2164.1747	583.2401
C97H163N22O33	C22H43N6O6S3
M+H	M+H
0.12	0.84

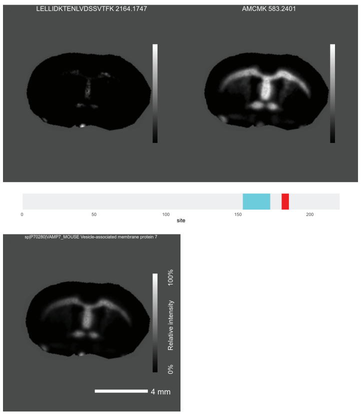

h Vesicle-associated membrane protein 8

GENLDHLRNK	KFWWK
1195.6178	794.4348
C49H83N18O17	C43H56N9O6
M+H	M+H
1.52	-0.32

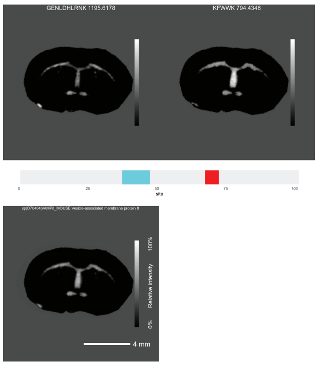

i Protein Kinase C eta type

GSFGKVMLAR	EFIWGVFGK	CQANVAPNCGVNAVELAK	ARFYAAEIISALMFLHEK	TLAGMGLQPGNISPTSKLISR	LGSLTQGGEHEILRHPFFK	MSSGTMK	FGINIPHK
1065.5874	1082.5669	1800.8731	2110.1154	2141.1747	2166.1454	741.3270	925.5254
C47H81N14O12S1	C55H76N11O12	C74H126N23O25S2	C99H153N24O25S1	C92H162N27O29S1	C99H153N28O27	C28H53N8O11S2	C44H69N12O10
M+H	M+H	M+H	M+H	M+H	M+H	M+H	M+H
-1.15	0.47	1.21	0.50	2.63	-0.86	1.24	0.52

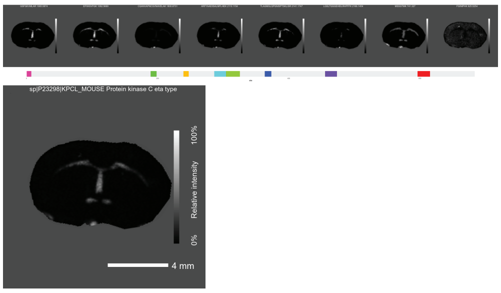

Supplementary Figure 4 cont.

j Smad nuclear interacting protein-1

SSDERPVSGQDR	VKPYIIDLGSGNGTFLNNK	EVPVKEKPSFELSGALLEDTNTFR	HRGHSR	LVEYTR
1332.6138	2050.0968	2706.3985	749.3914	780.4250
C51H86N19O23	C93H149N24O28	C121H193N30O40	C29H49N16O8	C35H58N9O11
M+H	M+H	M+H	M+H	M+H
0.23	0.98	3.02	0.40	1.84

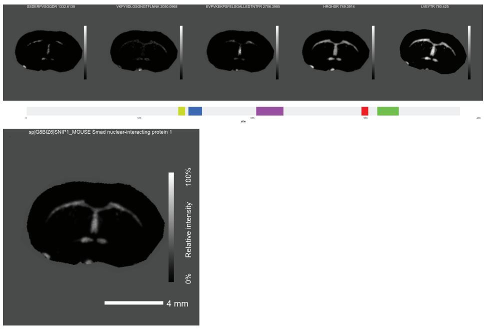

k Cytochrome c oxidase subunit 6A1

HEEHERPPFVAYPHLR	MASAVLSASR
2014.0042	992.5193
C92H133N28O24	C40H74N13O14S1
M+H	M+H
0.70	0.15

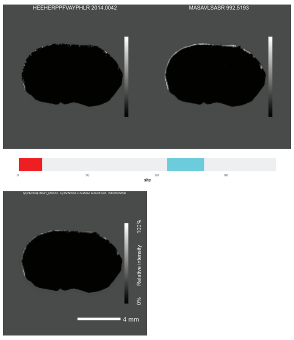

l Pyruvate Kinase

TVWVDYHNITQVVAVGGR	IGPEGLVTEVEHGGFLGNRK	GDAKTVWVDYHNITQVVAVGGR	DALGPEGR	QLFEELR	GVFPLLYR
2014.0505	2109.1087	2385.2310	814.4054	934.4993	964.5615
C91H141N26O26	C93H150N27O29	C106H166N31O32	C33H56N11O13	C42H68N11O13	C48H74N11O10
M+H	M+H	M+H	M+H	M+H	M+H
0.75	2.78	-0.48	-0.57	1.03	1.27

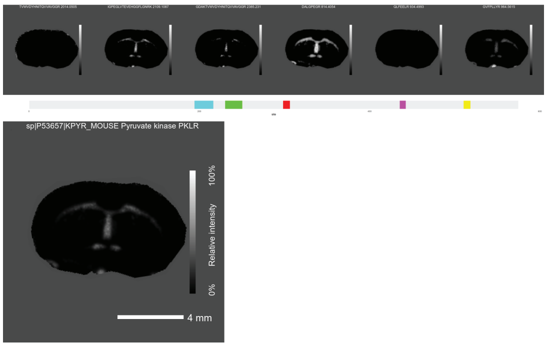

m Neutrophilic Granule Protein

AIEAYNQGRQGRPLFR	NIYEDAKYDIIGNILK
1875.994	1881.996
C82H131N28O23	C86H137N20O27
M+H	M+H
1.19	1.27

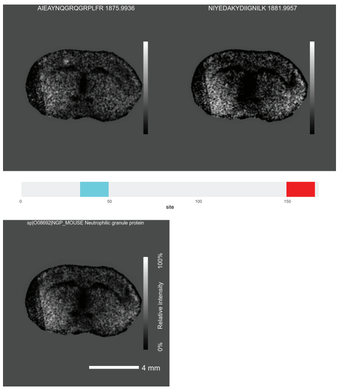

n Small EDRK rich factor 2

MTRGNQR	GNQRELAR
862.4312	943.5068
C32H60N15O11S1	C37H67N16O13
M+H	M+H
1.45	2.78

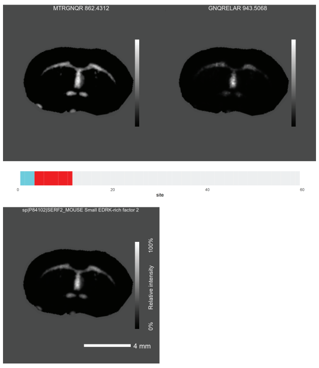

o Disintegrin and metalloproteinase domain-containing protein 22

QCKYIWGQK	EQRQLPQGDYVK	YIELMIVNDHLMFKK	LWETSI
1153.5823	1460.7492	1893.9965	748.3876
C53H81N14O13S1	C63H102N19O21	C88H141N20O22S2	C35H54N7O11
M+H	M+H	M+H	M+H
1.39	2.27	0.30	1.76

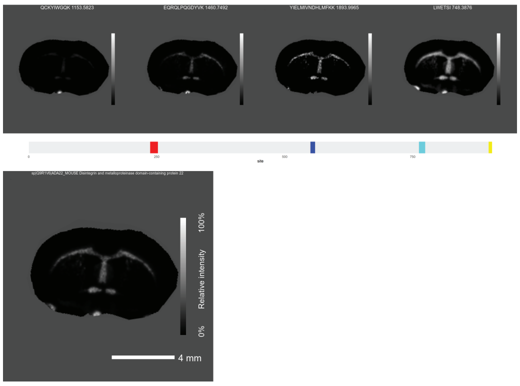

p Hyaluronan and proteoglycan link protein 3

YDVFCFAAALKGR	SFGDYQGR
1460.7355	929.4112
C68H102N17O17S1	C40H57N12O14
M+H	M+H
2.34	1.22

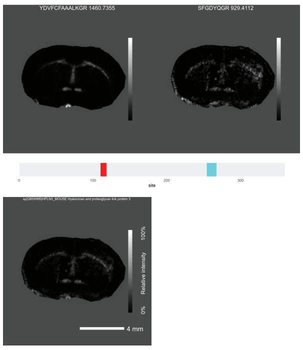

q Wnt-2b

ALMNLHNNR	QGYRHATR
1082.5524	988.5071
C44H76N17O13S1	C41H66N17O12
M+H	M+H
0.27	2.07

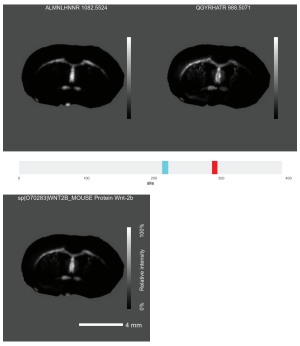

Supplementary Figure 4 | HIT-MAP analysis of murine brain. All peptide and protein cluster imaging outputs of annotated **a-c** neural receptors, **d-h** vesicle related proteins, **I** and **j** signalling proteins, **k** and **l** metabolic proteins, **m** and **n** Alzheimer's disease related proteins, and **o-q** extracellular proteins, scale bars = 4 mm. Intensity scales represent relative intensity from 0% to 100%.

Supplementary References

1. Wang, Z., Ryan, D. J. & Schey, K. L. Localization of the lens intermediate filament switch by imaging mass spectrometry. *Exp. Eye Res.* 108134 (2020). doi:10.1016/j.exer.2020.108134
