# Supplementary material for: Automated annotation and visualisation of high-resolution spatial proteomic mass spectrometry imaging data using HIT-MAP
Source: Nat Commun. 2021 May 28;12:3241. doi: 10.1038/s41467-021-23461-w (PMC8163805; doi:10.1038/s41467-021-23461-w)
Supplement: Supplementary file 2 — Reporting Summary [file 41467_2021_23461_MOESM2_ESM.pdf]

nature research

Corresponding author(s): Thomas R. Cox
Angus C. Grey

Last updated by author(s): Apr 12, 2021

Reporting Summary

Nature Research wishes to improve the reproducibility of the work that we publish. This form provides structure for consistency and transparency in reporting. For further information on Nature Research policies, see our Editorial Policies and the Editorial Policy Checklist.

Statistics

For all statistical analyses, confirm that the following items are present in the figure legend, table legend, main text, or Methods section.

n/a	Confirmed	
☐	☒	The exact sample size (*n*) for each experimental group/condition, given as a discrete number and unit of measurement
☒	☐	A statement on whether measurements were taken from distinct samples or whether the same sample was measured repeatedly
☒	☐	The statistical test(s) used AND whether they are one- or two-sided<br>*Only common tests should be described solely by name; describe more complex techniques in the Methods section.*
☐	☒	A description of all covariates tested
☐	☒	A description of any assumptions or corrections, such as tests of normality and adjustment for multiple comparisons
☒	☐	A full description of the statistical parameters including central tendency (e.g. means) or other basic estimates (e.g. regression coefficient) AND variation (e.g. standard deviation) or associated estimates of uncertainty (e.g. confidence intervals)
☐	☒	For null hypothesis testing, the test statistic (e.g. *F*, *t*, *r*) with confidence intervals, effect sizes, degrees of freedom and *P* value noted<br>*Give P values as exact values whenever suitable.*
☒	☐	For Bayesian analysis, information on the choice of priors and Markov chain Monte Carlo settings
☒	☐	For hierarchical and complex designs, identification of the appropriate level for tests and full reporting of outcomes
☒	☐	Estimates of effect sizes (e.g. Cohen's *d*, Pearson's *r*), indicating how they were calculated

Our web collection on statistics for biologists contains articles on many of the points above.

Software and code

Policy information about availability of computer code

Data collection

For data collection, Peptide calibrant spot imaging data were acquired using a SolariX XR 7T FT-ICR mass spectrometer equipped with a dual MALDI/ESI source and a dynamically harmonized ParaCell (Bruker Daltonics, Billerica, MA) and operated using ftmsControl v.2.2. Data were collected in positive ion mode from m/z 150–5000 with a resolving power (m/Δm) of 99,000 at m/z 400. Tandem mass spectrometry was performed using an isolation window of 3 or 10 Da to maximize signal without interference from other ions, and the collision energy was set to 40V.

Tissue imaging experiments were performed using a Bruker SolariX 15T FT-ICR mass spectrometer (Bruker Daltonics, Billerica, MA, USA) equipped with a dual MALDI/ESI source and a dynamically harmonized ParaCell (Bruker Daltonics, Billerica, MA) and operated using ftmsControl v.2.2. The MALDI source employs a Smartbeam II Nd:YAG laser system (2 kHz, 355 nm). Data were collected in positive ion mode from m/z 500-3000 with a resolving power (m/Δm) of 80,000 at m/z 1046.542 for bovine lens data and m/z 500-3000 with a resolving power of 60,000 for mouse brain data A raster step size of 150µm was used for lens data and 50 µm was used for brain data.

Data analysis

Data analysis was carried out using HIT-MAP which is open source and will be made freely available for download from https://github.com/MASHUOA/HiTMaP alongside user documentation prior to publication. HIT-MAP uses R (v3.6.2 or later) along with the following packages: Cardinal, Cleaver, Biostrings, protViz, rcdk, rcdklibs, FTICRMS, OrgMassSpecR,BiocParallel, colortools, Magick, knitr, dbplyr, xml2, stringr, ggplot2, reticulate, rJava, ncdf4, tibble, purrr and rcpp.

For manuscripts utilizing custom algorithms or software that are central to the research but not yet described in published literature, software must be made available to editors and reviewers. We strongly encourage code deposition in a community repository (e.g. GitHub). See the Nature Research guidelines for submitting code & software for further information.

Data

Policy information about availability of data

All manuscripts must include a data availability statement. This statement should provide the following information, where applicable:

- Accession codes, unique identifiers, or web links for publicly available datasets
- A list of figures that have associated raw data
- A description of any restrictions on data availability

Mass Spectrometry data pertaining to the Bruker peptide calibrant are available from the corresponding authors upon reasonable request. The data pertaining to the lens bovine tissue and mouse brain tissue mass spectrometry imaging datasets have been deposited to the ProteomeXchange Consortium [http://proteomecentral.proteomexchange.org] via the PRIDE partner repository with the dataset identifier PXD025486. All other relevant data supporting the findings are available within the paper and its supplementary information files.

Field-specific reporting

Please select the one below that is the best fit for your research. If you are not sure, read the appropriate sections before making your selection.

☒ Life sciences ☐ Behavioural & social sciences ☐ Ecological, evolutionary & environmental sciences

For a reference copy of the document with all sections, see nature.com/documents/nr-reporting-summary-flat.pdf

Life sciences study design

All studies must disclose on these points even when the disclosure is negative.

Sample size	Biological replicates are not relevant to the computational pipeline of HIT-MAP, so n=1 for each tissue type analysed
Data exclusions	No data exclusion undertaken
Replication	Replication was not required as the HIT-MAP computational pipeline runs specifically on individual datasets and not replicates
Randomization	No randomisation performed or required in this study
Blinding	No blinding of researchers to datasets was undertaken, however HIT-MAPs computational pipeline treats all datasets the same

Reporting for specific materials, systems and methods

We require information from authors about some types of materials, experimental systems and methods used in many studies. Here, indicate whether each material, system or method listed is relevant to your study. If you are not sure if a list item applies to your research, read the appropriate section before selecting a response.

Materials & experimental systems

n/a	Involved in the study	
☒	☐	Antibodies
☒	☐	Eukaryotic cell lines
☒	☐	Palaeontology and archaeology
☐	☒	Animals and other organisms
☒	☐	Human research participants
☒	☐	Clinical data
☒	☐	Dual use research of concern

Methods

n/a	Involved in the study	
☒	☐	ChIP-seq
☒	☐	Flow cytometry
☒	☐	MRI-based neuroimaging

Animals and other organisms

Policy information about studies involving animals; ARRIVE guidelines recommended for reporting animal research

Laboratory animals	No laboratory animals used - mouse tissues were purchased from Pel-Freez for analysis
Wild animals	No wild animals used
Field-collected samples	No field collected samples used
Ethics oversight	No ethics required as mouse tissues were purchased direct from Pel-Freez for analysis.

Note that full information on the approval of the study protocol must also be provided in the manuscript.
